# Improvement of Artificial Hummingbird Algorithm with Greedy Fitness–Distance Balance Method and Its Application to Load Frequency Control

**DOI:** 10.3390/biomimetics11100754

**Published:** 2026-10-19

**Authors:** Tural Aslanov, Bora Çavdar, Ömür Akyazı, Kenan Yanmaz

**Affiliations:** 1 Department of Energy Systems Engineering, Karadeniz Technical University, 61830 Trabzon, Türkiye; 412267@ogr.ktu.edu.tr (T.A.); boracavdar@ktu.edu.tr (B.Ç.); 2 Bulancak Vocational School, Giresun University, 28300 Giresun, Türkiye; kenan.yanmaz@giresun.edu.tr

**Keywords:** artificial hummingbird algorithm, fitness–distance balance, performance improvement of metaheuristic algorithms, load frequency control

## Abstract

This paper proposes an improved Artificial Hummingbird Algorithm (AHA) for solving numerical optimization problems. The greedy Fitness–Distance Balance (FDB) selection method is integrated into the guided, territorial, and migration search operators of the AHA. This establishes a balance between exploration and exploitation by enhancing AHA’s exploration capabilities. The performance of the proposed FDB-AHA is evaluated using CEC 2017 and 2020 problems which include single-mode, multi-mode, hybrid, and composition functions in search spaces of dimensions 30, 50, and 100. Furthermore, the results of the FDB-AHA are compared with 11 other advanced search algorithms using the Friedman statistical test method on the CEC 2017 and 2020 problems. As a result of these comparisons, the FDB-AHA was found to be the most effective method, while the AHA ranked ninth. These findings confirm the effectiveness of the improvements made in the AHA. Moreover, the FDB-AHA has been used to determine the optimal solutions for load frequency control in three different power systems. In this context, three-term (PID), five-term (FOPID), and six-term (FOPI-FOPD) controllers are optimized and compared with existing studies in the literature for time-domain analysis. Furthermore, load variation and robustness analysis are also performed. Finally, the practicality of the controllers optimized by FDB-AHA is verified using the real-time simulator OP5707. Simulation results show that FDB-AHA is a highly efficient algorithm for solving all optimization problems.

## 1. Introduction

Optimization is the process of obtaining the optimum or near-optimal solution of a problem under specific constraints. The objective throughout this process is to obtain the maximum or minimum value, in other words, to decrease or increase the solution throughout the steps of the process [1]. Optimization methods can be generally divided into two categories. The first one is deterministic methods, which are devoid of uncertainty and randomness, while the second one is stochastic methods including heuristic search techniques [2]. Both methods involve specific designs for the problem for which an optimum solution is desired. The concept of metaheuristics can be defined as a more advanced version of heuristics, intended to provide a more general approach to a problem rather than a specific approach [3]. Metaheuristic search (MHS) algorithms were initially not very popular due to their high computational complexity but have become widespread in the literature thanks to advances in technology.

MHS algorithms with iterative phases generally have two basic functions: exploration and exploitation. While the exploration function objectives to increase the diversity of the solution candidates during the search, the exploitation function is expected to improve the neighborhood of the solution candidates. MHS algorithms maintain a good balance between exploration and exploitation throughout iteration, preventing local optima stagnation and premature convergence. MHS algorithms with similar objectives and phases have different designs. These designs can be considered depending on the inspiration of the design. If MSH algorithms are classified according to their design inspiration, they can be comprehensively categorized into four classes. These classes can be expressed as “inspired by swarm intelligence”, “inspired by evolutionary processes”, “inspired by physical and mathematical models”, and “inspired by related fields of human behavior” [4]. Swarm-intelligence-inspired MHS algorithms include Particle Swarm Optimization (PSO), Cuckoo Search (CS), the Bat Algorithm (BA), and Grey Wolf Optimization (GWO); evolutionary-process-inspired MHS algorithms include the Genetic algorithm (GA), Differential Evolution (DE), and Stochastic Fractal Search (SFS); human-behavior-inspired MHS algorithms include Teaching–Learning-Based Optimization (TLBO), the Human-Inspired (HI) algorithm, the League Championship (LC) algorithm, and the Exchange Market (EM) algorithm; physical and mathematical model inspired MHS algorithms include the Gravitational Search (GS) algorithm, Charged System Search (CSS), the Black Hole (BH) algorithm, and the Sine Cosine (SC) algorithm.

In addition to the design of new MHS algorithms, the improvement of existing algorithms has become a significant area of research in recent years. These studies can generally be categorized into three groups: dynamic parameter tuning, hybrid algorithm design, and candidate solution selection methods.

Dynamic parameter tuning is one of the key improvement approaches used to modify the search behavior of existing algorithms. This approach utilizes self-adaptive parameters, chaotic maps, Levy flight, and similar mechanisms [5,6,7,8,9,10]. In [5], SA-BO was developed by adding self-adaptive parameters based on repulsion-based learning to the Bonobo Optimization (BO) algorithm. In [6], the constant parameter structure of the CS algorithm was modified by introducing adaptive parameter control based on the Cauchy distribution and the Lehmer mean, resulting in A-CS. In [7], the Phasmatodea Population Evolution (PPE) algorithm was extended by using chaotic maps for the initial population to create Chaotic PPE (C-PPE). While in [8], the SC algorithm was extended by using the Singer Map to determine the initial solution candidates, resulting in Chaotic SC (C-SC). In [9], the normal distribution used for solution candidate selection in the Ant Colony Optimization (ACO) algorithm was replaced with Levy flight, resulting in Levy ACO (L-ACO). In [10], Golden Sine (GS) operators and the Levy flight mechanism were added to the BH algorithm, leading to the development of L-GS-BH.

Another improvement approach involves hybrid algorithm designs that combine specific mechanisms from different algorithms [10,11,12,13,14,15,16]. In [11], ACO-BSO was developed by combining the pheromone-based learning mechanism of ACO with the dynamic position update mechanism of Bird Swarm Optimization (BSO). In [12], the Aquila Optimization (AO) and Arithmetic Optimization (ArO) algorithms were combined in an equal-probability solution update structure to create AO-ArO. The authors of [13] combine the exploitation mechanism of GWO with the exploration mechanism of Harris Hawks Optimization (HHO) to develop GWO-HHO. In [14], the exploitation mechanism of HHO and the exploration mechanism of Moth-Flame Optimization (MFO) were combined with chaotic maps and Evolutionary Population (EP) dynamics to create EP-C-HHO-MFO. In [15], the initial population generated by PSO was continued using the Sparrow Search (SS) algorithm to develop PSO-SS. While in [16], the initial population generated by Simulated Annealing (SA) was continued using Manta Ray Foraging Optimization (MRFO) to develop SA-MRFO.

Another approach used for improving MHS algorithms involves modifying candidate selection methods or integrating different selection mechanisms into the algorithms. For this purpose, the Greedy (G), Roulette Wheel (RW), and Tournament (T) methods, as well as their variants, are used. As an alternative to these, the Fitness–Distance Balance (FDB) method, which evaluates both the fitness value and the distance to the best solution, has been developed [17]. Studies in this field can be exemplified by [18,19,20,21,22,23,24,25]. In [18], the LPM-RW-E algorithm was developed by adding a Level-based Population Model (LPM) and RW to the Evolutionary algorithm. In [19], the random tree selection in the seed generation phase of the Tree Seed (TS) algorithm was replaced with RW to create RW-TS. In [20], the Antlion Optimization (AnO) algorithm was enhanced by adding T-selection to its random walk phase, resulting in T-AnO. In [21], the roulette wheel-based selection in the Fireworks Optimization (FO) algorithm was replaced with tournament selection, resulting in T-FO. In [22], the Epsilon-Greedy (EG) selection method was added to the ACO algorithm, and EG-L-ACO was developed by using Levy flight in the randomization phase. In [23], Greedy Crossover (GC) was added to the solution generation process of the Bat algorithm, resulting in GC-B. In FDB-based studies, in [24], the FDB method was integrated into the decomposition phase of the Artificial Ecosystem Optimization (AEO) algorithm, and RW-FDB-AEO was developed by using RW to select the candidates obtained via FDB. In [25], FDB was integrated into the mutation phase of the Adaptive Guided DE (AG-DE) algorithm, and RW was used to select the resulting candidates, leading to the development of RW-FDB-AG-DE. In [26], the FDB method was integrated into the guidance mechanism of the Weighted Chimp Optimization Algorithm (WChOA) to develop FDB-WChOA. In [27], the FDB and dynamic FDB (dFDB) methods were integrated into the guide selection mechanism of the Starfish Optimization Algorithm (SFOA), resulting in FDBSFOA and dFDBSFOA.

The algorithm improvement studies discussed above are summarized in Table 1. The table lists, for each study, the type of improvement, the original algorithm, the modification applied, the improved algorithm, and the problem addressed.

The literature shows that in algorithm improvement studies, researchers aim to improve and balance the exploration and exploitation phases of algorithms by using different methods. The algorithms obtained following the improvements are tested on mathematical problems and engineering problems to demonstrate their performance. This study aims to improve the Artificial Hummingbird Algorithm (AHA) using the FDB method. In this study, the performance of the proposed FDB-AHA is tested on the proven CEC benchmark problems (CEC2017, CEC2020), which are frequently used in algorithm improvement studies in the literature. In addition, FDB-AHA is also tested on the Load Frequency Control (LFC) problem, which is an important and popular topic in the field of power systems and control systems and compared with studies in the literature. LFC is required to dampen frequency oscillations during sudden load variation in power systems. LFC has become more challenging with the change in load types, increase in consumption, and integration of new generation sources in power systems. In particular, the increasing integration of grid-connected photovoltaic systems requires control strategies capable of addressing grid-interfacing requirements [28]. To overcome this challenge, the LFC problem in different power systems has been frequently considered by researchers. In the studies considered, the controllers used for the LFC problem are optimized with MHS algorithms. In these studies, original algorithms as well as improved algorithms are used for optimization. Examples of these algorithms used in LFC problem are Black Widow Optimization (BWO) [29], GWO-CS [30], the Dragonfly Search (DS) algorithm [31], Jellyfish Search Optimizer (JSO) [32], the Lion Algorithm (LA) [33], Harmony Search-CS (HS-CS) [34], TLBO [35], Improved-ACO (I-ACO) [36], Local Unimodal Sampling-TLBO (LUS-TLBO) [37], I-GWO [38], SFS-Pattern Search (SFS-PS) [39], Shuffled Frog-Leaping-PS (SFL-PS) [40], the I-Whale Optimization (I-WO) algorithm [41], the Cohort Intelligence Optimization (CIO) algorithm [42], the Levy-Imperialist Competitive (L-IC) algorithm [43], GA [44], PSO [44], and DE [44,45]. The algorithms given as examples are generally used to optimize the parameters of controllers such as Proportional–Integral–Derivative (PID), Fractional-Order PID (FOPID), tilt-ID (TID), and Fuzzy-PID. In the optimization process, the Integral of Time-Weighted Absolute Error (ITAE) is generally used as the objective function. Although not frequent, Integral of Squared Error (ISE), Integral of Absolute Error (IAE), and Integral of Time-Weighted Squared Error (ITSE) objective functions have also been considered. Table 2 provides a summary of the studies addressing the LFC problem, including the power system structures considered and the corresponding number of areas.

This paper presents an improved version of the AHA inspired by the behavior of hummingbirds during foraging. In this version, the exploration capability and the balance between exploration and exploitation of the AHA have been improved. These improvements are made by integrating the FDB method into foraging operators of the AHA. The proposed FDB-AHA is compared with the AHA and 10 other well-known MHS algorithms using Friedman and Wilcoxon statistical tests on the benchmark problems CEC2017. The performance of the FDB-AHA in LFC is then used to optimally design the parameters of three different controllers (PID, FOPID, FOPI-FOPD) in three different power systems (Thermal/Thermal, Thermal/Hydro/Gas, and PV/Thermal). The obtained optimal designs are compared with the studies in the literature using the same power systems. In addition, the resulting optimal controller designs were tested under load variation and robustness analysis. Finally, the performance of the optimal controller designs obtained is verified using the OPAL-RT real-time simulator. The main contributions of this study to literature can be summarized as follows:

- The main innovation of this study is the integration of the FDB selection mechanism into all three fundamental foraging operators of AHA (guided, territorial, and migration) in a manner specific to each operator and dependent on the iteration process. Thus, FDB has been employed within a comprehensive framework that contributes to all the fundamental operators constituting AHA’s foraging behavior, rather than merely at a specific selection or update point, while the candidates selected by the FDB are used in different ways during the two halves of the optimization process in the guided foraging phase, in the territorial foraging phase it is applied only in the first quarter to guide local search toward qualified candidates, and in the migration foraging phase, the random repositioning in the standard AHA is enhanced with FDB-based selection. This comprehensive development has elevated the standard AHA’s overall Friedman ranking for the CEC 2017 and CEC 2020 problems from ninth to first place among 12 algorithms.
- The developed FDB-AHA is compared with the AHA and 10 other well-known MHS algorithms in the literature using Friedman and Wilcoxon test methods on CEC 2017 and 2020 problems. In addition, the performances of the FDB-AHA and the AHA are also compared with box plots and convergence plots.
- With the improved FDB-AHA, three different controllers (PID, FOPID, FOPI-FOPD) for load frequency control in Thermal/Thermal, Thermal/Hydro/Gas, and PV/Thermal power systems are optimally designed using the ITAE objective function. The optimum controller designs obtained are compared with the optimum controller designs in the literature and the superior performance of the FDB-AHA is proved.
- The success of the obtained optimal control designs has been demonstrated through load variation and robustness analyses.
- The OPAL-RT OP5707 real-time simulator is utilized to validate the obtained LFC results, ensuring the practical feasibility of the proposed approach.
- A comprehensive review and classification of recent MHS algorithm improvement strategies are presented in Section 1. This review-oriented introduction provides researchers with a comprehensive overview of the historical evolution, current trends, and recent advancements in MHS algorithm development.

## 2. Methodology

### 2.1. Artificial Hummingbird Algorithm

The AHA is an MHS algorithm inspired by the foraging strategies of hummingbirds in nature. A hummingbird has three different flight skills: axial, diagonal and multidirectional. Moreover, their memory ability to select the target food source is the most important component of the AHA. AHA mimics three different foraging behaviors, namely guided, territorial, and migration foraging, during the optimization process [46].

Initialization: In this subsection, the basic architecture of AHA is briefly explained mathematically. Within the allowed upper and lower bounds, the random starting positions of the hummingbirds are determined as in Equation (1).

(1) $$x_{i}=\mathrm{Low}+r\;\cdot \;\left(\mathrm{Up}-\mathrm{Low}\right),\;i=1,\ldots ,n$$

Visit Table: Once the starting positions of the hummingbirds (which are also used as initial food sources) are determined, the visit table, one of the unique parts of the algorithm, is initialized. The visit table keeps track of how many iterations a hummingbird has not visited each food source In the table, the food source that has not been visited for the longest time has the highest value. Therefore, in the next iteration, the hummingbird moves toward the food source with the highest value in the table. If there are food sources with the same high value at different positions in the visit table, the source with the best fitness value is selected first. The visit table is updated after each foraging stage. The visit table is initialized as in Equation (2).

(2) $$VT_{i,j}=\left\{\begin{array}{cc} 0\;\mathrm{if}\; & i\neq j\\ \;\mathrm{null} & i=j \end{array}\right.\;i=1,\ldots ,n\;\;j=1,\ldots ,n$$

In Equation (2), if i=j, the hummingbird is already in its own position, so the number of visits is not tracked. When the bird visits a location, the number of visits is reset to zero, while the number of other locations is incremented by one. Furthermore, the food source is updated if the food source visited by the hummingbird is better than its previous food source.

Flight Techniques: A hummingbird can fly toward a target food source using three different flight techniques. These techniques are axial, diagonal, and omnidirectional flight. These three flight techniques facilitate hummingbirds when foraging, feeding, and avoiding predators. The difference from other birds is that a hummingbird is skilled in axial and diagonal flight. In the AHA, a change of direction vector is modeled. This vector decides which flight technique a hummingbird will use to reach the target food source in a *d*-dimensional search space. The mathematical models of axial, diagonal, and omnidirectional flight are given in Equation (3), Equation (4) and Equation (5), respectively.

(3) $$D^{\left(i\right)}=\left\{\begin{array}{cc} 1\;\mathrm{if}\; & i=\mathrm{randi}([1,d])\\ 0\;\mathrm{else} & \end{array}\right.\;\;i=1,2,\ldots ,d$$

(4) $$D^{\left(i\right)}=\left\{\begin{array}{cc} 1\;\mathrm{if}\; & i=P(j),\;j\in [1,k],\;P=\mathrm{randperm}(k)\\ \; & k\in \left[2,\lceil r_{1}\;\cdot \;\left(d-2\right)\rceil +1\right]\\ 0\;\mathrm{else} & \end{array}\right.\;i=1,2,\ldots ,d$$

(5) $$D^{\left(i\right)}=1,\;i=1,2,\ldots ,d$$

where randi([1,d]) is a vector of integers from 1 to *d*, randperm(k) generates a random permutation of integers from 1 to *k*, and $r_{1}$ is a random number between 0 and 1.

Guided Foraging: In the AHA, each hummingbird identifies its target food source from the visit table and plans to move towards it. The target food source is the one with the highest value. The mathematical model of guided foraging is given in Equation (6).

(6) $$v_{i}\left(t+1\right)=x_{i,\mathrm{tar}}\left(t\right)+a\;\cdot \;D\;\cdot \;\left(x_{i}\left(t\right)-x_{i,\mathrm{tar}}\left(t\right)\right)$$

where $x_{i}\left(t\right)$ is the location of the food source where the *i*-th hummingbird is located at time *t*, $x_{i,\mathrm{tar}}\left(t\right)$ is the location of the target food source that the *i*-th hummingbird plans to visit, and *a* is the normally distributed guide search factor.

Territorial Foraging: The territorial foraging stage is the stage where each hummingbird visits food sources in its neighborhood. At this stage, territorial search is performed, and if a food source with a higher value than the current one is found, the location of the food source is updated. The mathematical model of territorial foraging is given in Equation (7) where *b* is the territorial search factor with a normal distribution.

(7) $$v_{i}\left(t+1\right)=x_{i}\left(t\right)+b\;\cdot \;D\;\cdot \;x_{i}\left(t\right)$$

Migration Foraging: In the AHA, if a hummingbird’s food source is insufficient, it migrates to other sources in different regions. At this stage, a migration coefficient is set. If the number of iterations exceeds the value of the migration coefficient, the hummingbird at the food source with the least food migrates to a different region. The mathematical model of foraging migration is given in Equation (8).

(8) $$x_{\mathrm{wor}}\left(t+1\right)=\mathrm{Low}+\mathrm{rand}\;\cdot \;\left(\mathrm{Up}-\mathrm{Low}\right)$$

where Low and Up are the lower and upper bounds for a *d*-dimensional problem, $x_{\mathrm{wor}}$ is the location of the food source with the worst nectar filling rate, and rand is a random number between 0 and 1.

### 2.2. Greedy Fitness–Distance Balance

The recently proposed FDB method is designed to improve the search performance of MHS algorithms [17]. This method evaluates the solution candidates in the population based on their fitness values and their distance from the best solution candidate. As a result of this evaluation, all solution candidates are scored. A solution candidate that has both a low fitness value and is far from the best solution candidate receives the highest score. The solution candidates that are close to the best solution candidate have low fitness values but low scores. In this way, high-scoring solution candidates contribute to diversity (exploration) within the existing population, helping to overcome the shortcomings of the best solution candidate. As a result, the FDB method can achieve more successful results by balancing exploration and exploitation.

The FDB method can be applied in a few steps. First, the fitness values $\left(F_{P}\right)$ of the solution candidates are determined. $F_{P}$ is determined according to the values obtained from the objective function of the problem as in Equation (9).

(9) $$F_{P}\equiv {\left[\begin{array}{c} f_{1}\\ \vdots \\ f_{n} \end{array}\right]}_{n\times 1}$$

Then, the distance vector between each solution candidate and the best solution candidate is created to calculate the FDB scores. In a population *P* with *m* design variables and *n* solution candidates, the Euclidean distance between the *i*-th solution candidate $\left(x_{i}\right)$ and the best solution candidate $x_{\mathrm{best}}$ is calculated as in Equation (10) [17].

(10) $${i{=}_{1}}^{n}\forall P_{i},D_{Pi}=\sqrt{{\left(x_{1_{i}}-x_{1_{\mathrm{best}}}\right)}^{2}+{\left(x_{2_{i}}-x_{2_{\mathrm{best}}}\right)}^{2}\cdots +{\left(x_{m_{i}}-x_{m_{\mathrm{best}}}\right)}^{2}}$$

The distance vector $\left(D_{P}\right)$ is expressed as in Equation (11).

(11) $$D_{P}\equiv {\left[\begin{array}{c} d_{1}\\ \vdots \\ d_{n} \end{array}\right]}_{n\times 1}$$

The FDB score of each solution candidate with the determined fitness value and distance value is calculated as shown in Equation (12). In the score calculation, the values for fitness and distance are normalized to prevent one from dominating the other. In addition, a weight coefficient (w) is used to determine the effect of $F_{P}$ and $D_{P}$ values in the equation. In this study, *w* is selected as 0.5 by referring to [17].

(12) $${i{=}_{1}}^{n}\forall P_{i},S_{Pi}=w\;\cdot \;{\mathrm{normF}}_{Pi}+\left(1-w\right)\;\cdot \;{\mathrm{normD}}_{Pi}$$

As a result, the FDB score vector $\left(S_{P}\right)$ is expressed as in Equation (13). In Equation (13), $S_{1}$ is the score of the best solution candidate according to the FDB method. Using this solution candidate obtained in the FDB method is a greedy selection.

(13) $$S_{P}\equiv {\left[\begin{array}{c} s_{1}\\ \vdots \\ s_{n} \end{array}\right]}_{n\times 1}$$

### 2.3. Greedy Fitness–Distance Balance-Based Artificial Hummingbird Algorithm

Inspired by the feeding and fast-moving strategies of hummingbirds in nature, the AHA can provide fast and highly successful solutions to complex and multidimensional optimization problems. In the AHA, hummingbirds have three different flight techniques and foraging operators. These flight techniques and foraging operators contribute to the exploration and exploitation phases of the algorithm. The contributions of the algorithm’s flight techniques and foraging operators to the algorithm’s exploration and exploitation phase can be summarized as follows.

- While the axial flight technique contributes more to the exploitation phase of the algorithm than other flights, the omnidirectional flight technique contributes the most to the exploration phase of the algorithm.
- In the guided foraging operator, the randomly determined coefficient a strengthens the exploitation phase of the algorithm at small values and strengthens the exploration phase at large values.
- The territorial foraging operator contributes more to the exploitation phase compared to the guided foraging operator. Furthermore, as the value of the coefficient *b* in this operator increases, the transition from exploitation to exploration can be achieved.
- The migration foraging operator contributes to exploration by replacing the worst result with a random one but does not contribute much to exploitation.
- While the visit table contributes to the exploration phase by directing the solution candidate to a resource that has not been visited for a long time, if two resources have not been visited for the same amount of time, it contributes to the exploitation phase by directing the solution candidate to the one with a better fitness value.

It can be said that the techniques and operators used in the AHA (as mentioned above) contribute to both exploration and exploitation at the same time. This proves that a good balance between exploration and exploitation with the techniques and operators used in the algorithm is very effective in the success of the algorithm. However, it has been observed that the AHA cannot provide stability in different runs and sizes in solving the CEC 2017 and 2020 problems. In particular, analysis has shown that the performance of the AHA is lower in multimodal functions. It can be inferred that the algorithm has poor exploration properties and does not maintain the exploration–exploitation balance well. Therefore, this paper provides an improved version of the AHA, which has been redesigned with the FDB method to improve its exploration and exploitation capabilities.

In this study, the FDB method is integrated into the operators of the algorithm that contribute to the exploration and exploitation phases with different combinations. Afterwards, the algorithm is fine-tuned by trying many different variations of the algorithm operators developed with the FDB method, which are suitable for improvement. The proposed FDB-AHA is developed by integrating the FDB method into the guided, territorial, and migration search operators in the AHA. In this study, the algorithm’s progress is tracked based on the number of objective function evaluations. Therefore, the iteration rates and thresholds used below correspond to their respective values within the total objective function evaluation budget. The integration in the guided foraging operator is fine-tuned by splitting the iterative process into two equal parts. Equation (14) is used in the first part of the iteration process and Equation (15) in the second part. In this way, the guided foraging process of the algorithm searches both around the best solution candidate selected in terms of fitness value and around the solution candidate selected by the FDB method. In this way, the algorithm avoids monotonous progress, effectively improving the exploration process in the first part of the iteration process and creating a better balance between exploration and exploitation in the second part of the iteration.

(14) $$v_{i}\left(t+1\right)=x_{i,\mathrm{tar}}\left(t\right)+a\;\cdot \;D\;\cdot \;\left(x_{i}\left(t\right)-x_{i,\mathrm{FDB}}\left(t\right)\right)$$

(15) $$v_{i}\left(t+1\right)=x_{i,\mathrm{FDB}}\left(t\right)+a\;\cdot \;D\;\cdot \;\left(x_{i}\left(t\right)-x_{i,\mathrm{tar}}\left(t\right)\right)$$

In the territorial foraging operator, the FDB is used only during the first quarter of the optimization process. At this stage, the solution candidate selected using the FDB method is incorporated into the standard territorial foraging equation with the aim of directing the local search process toward higher-quality candidates. This ensures that high-quality solution regions are targeted more quickly in the early stages of the algorithm. After the first quarter of the maximum number of iterations, the standard territorial foraging operator continues to be used. The mathematical expression of this structure is given in Equation (16).

(16) $$v_{i}\left(t+1\right)=x_{i}\left(t\right)+b\;\cdot \;D\;\cdot \;x_{i,\mathrm{FDB}}\left(t\right)$$

While the migration foraging operator in the AHA generates a random solution candidate in the case mod(Iteration,2×TotalSolutionCandidate)=0 depending on the iteration process, in the improved version, this solution candidate is obtained using the FDB method as given in Equation (17). In this way, the exploration process is strengthened by incorporating both randomness and information, and the selection of a solution candidate based only on randomness is avoided.

(17) $$x_{\mathrm{wor}}\left(t+1\right)=x_{i,\mathrm{FDB}}\left(t\right)$$

**Figure 1:**
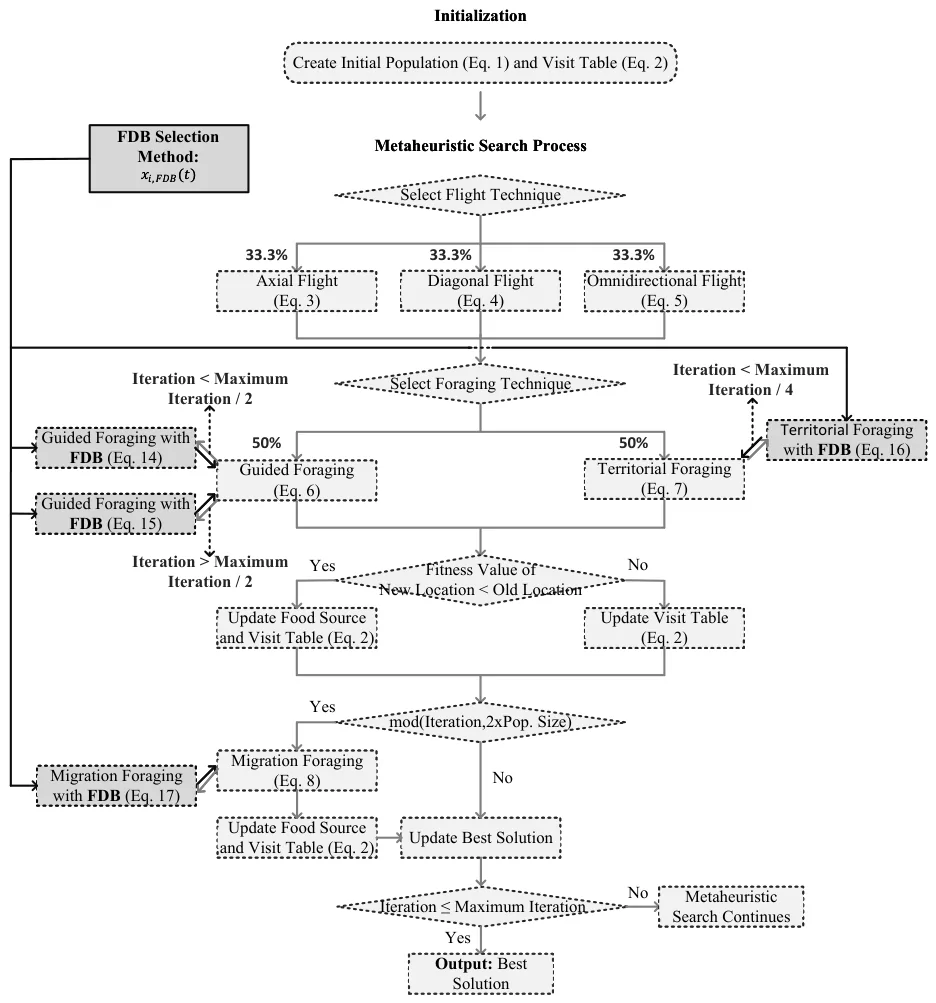
Flowchart of FDB-AHA.

Figure 1 shows the flowchart of the proposed FDB-AHA for a clearer understanding of the algorithm stages and the improvements implemented using the FDB method mentioned above.

The computational complexity of the proposed FDB-AHA has been determined by taking into account the FDB operation added to the standard AHA. Here, *M* denotes the maximum number of iterations, *N* denotes the population size, and *D* denotes the problem dimension. According to this definition, the computational complexity of the basic search operations in the standard AHA is approximately O(MD). Since the FDB operation calculates the distance of each candidate solution in the population from the best candidate solution across *D* dimensions, the computational complexity of a single FDB operation is O(ND). Because the FDB operation is applied during the update of each candidate solution, the additional cost for a population sweep is $O(N^{2}D)$. Since a population sweep involves approximately *N* solution updates, approximately M/N population sweeps are performed over the total *M* iterations, and the additional computational cost of FDB throughout the entire optimization process is approximately O(MND). Therefore, the total computational complexity of FDB-AHA can be expressed predominantly as O(MND).

### 2.4. Mathematical Problems Utilized as Objective Function

Competition in the ever-expanding list of algorithms is determined by benchmark functions presented at international CEC (IEEE Congress on Evolutionary Computation) conferences. In recent years, various optimization problems set in the CEC 2017 [47] and CEC 2020 [48] conferences define the standards for the competition between the developed algorithms. Furthermore, the performance of these solution methods is also tested by adapting them to more complex engineering problems.

In this study, the performance of the developed FDB-AHA is tested on CEC 2017 and 2020, which have a wide problem set and are frequently used in the literature. These functions are classified into four categories: unimodal, multimodal, hybrid, and composition. These functions evaluate the algorithms’ exploitation, exploration, balance between exploration and exploitation, and search capabilities in high-complexity search spaces, respectively. The CEC 2017 benchmark test functions list consists of 30 functions derived from classical test functions, including 3 unimodal (F1–F3), 7 multimodal (F4–F10), 10 hybrid (F11–F20), and 10 composition (F21–F30). The F2 function was removed from the CEC 2017 test suite due to its unstable behavior in high dimensions [47]. The CEC 2020 test function list consists of 10 functions derived from classical test functions, 1 unimodal (F1), 3 multimodal (F2–F4), 3 hybrid (F5–F7) and 3 composition (F8–F10) [48].

### 2.5. Power System Problem: Load Frequency Control

#### 2.5.1. Power Systems (Thermal/Thermal—Thermal/Hydro/Gas—PV/Thermal)

**Figure 2:**
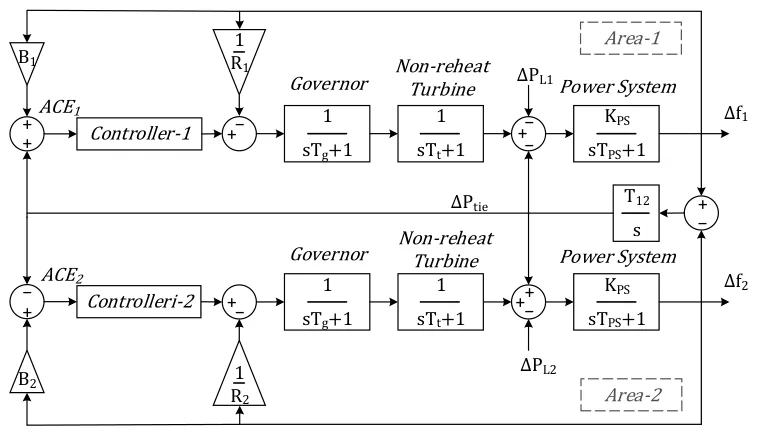
Two–area non-reheat thermal system model.

The linear model of the two-area non-reheat thermal system is shown in Figure 2. This model is one of the most widely used models in LFC studies in the literature. The model parameter values are provided in Appendix A and are based on previous studies [34,44,49].

**Figure 3:**
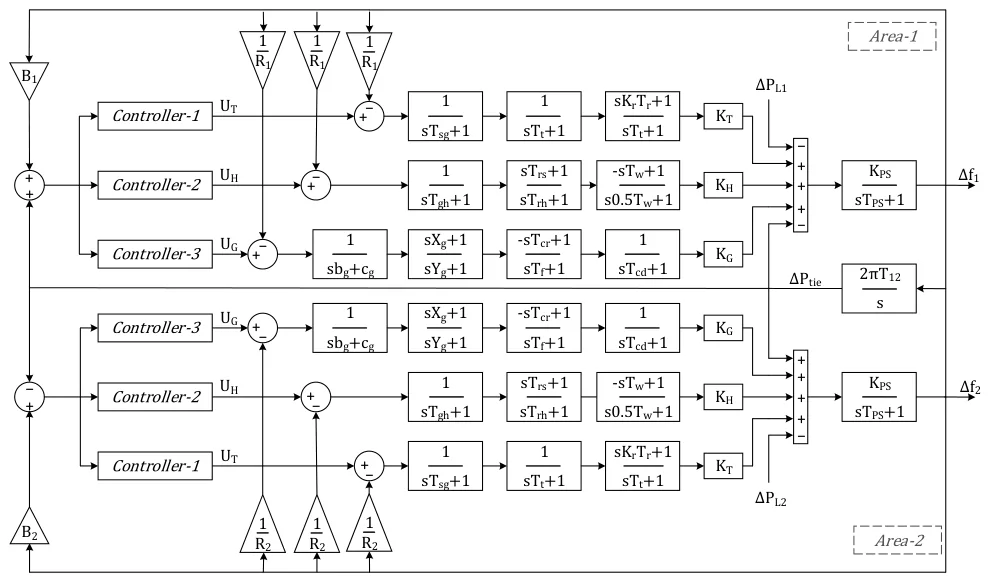
Two–area multi-source system model [31].

The second system examined in this study is a two-area multi-source system, which is also widely studied in the literature. In this system, there are three sources for each field. These are thermal, hydro, and gas units [31,45,49]. This system is shown in Figure 3. The system parameter values are provided in Appendix A.

**Figure 4:**
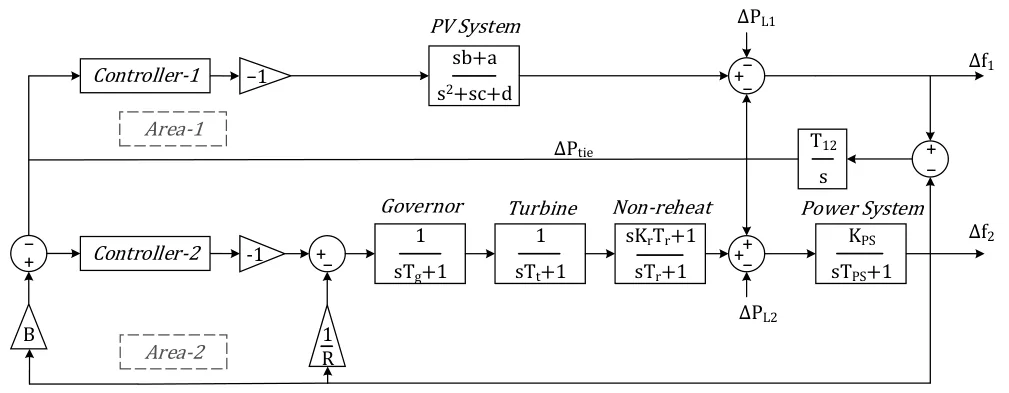
Two-area PV–thermal system model [40,41].

The third system is a two-area PV–thermal system that contains renewable energy sources, which is widely used in the relevant literature. This system is presented in Figure 4. The system parameter values are provided in Appendix A.

#### 2.5.2. Control Systems (PID/FOPID/FOPI-FOPD)

The PID controller, also known as a three-term controller, is widely used in the automatic control field due to its simple and stable structure [40,50]. The transfer function of the PID controller is given in Equation (18).

(18) $$G_{PID}\left(s\right)=\frac{U(s)}{E(s)}=K_{P}+\frac{K_{I}}{s}+K_{D}s$$

where U(s) and E(s) are the output and input signals of the controller in Laplace domain, respectively. U(s) is the control signal and E(s) is the error signal, which is the difference between the reference (R(s)) and actual output (Y(s)). $K_{P}$, $K_{I}$, and $K_{D}$ are the proportional, integral, and derivative gains, respectively.

Studies in the field of fractional mathematics have made it possible to perform fractional operations with derivative and integral operators. With this development, it is possible to convert integer classical PID controllers to fractional PID controllers. The most used method for fractional calculation, especially for control systems, is the Oustaloup convergence method [51,52]. The mathematical equation of the Oustaloup convergence method based on the bandpass filter is given in Equation (19).

(19) $$s^{\alpha }\approx \omega_{h}^{\alpha }\prod_{k=-N}^{N}\frac{s+\omega_{z}}{s+\omega_{p}},\;\mathrm{for}\;0<\alpha <1$$

where *N* is used to determine the filter order, and it is selected as 5 in this study. $\omega_{z}$ and $\omega_{p}$ represent the zeros and poles of the Oustaloup filter, respectively. Furthermore, the mathematical formulation of the improved Oustaloup convergence method is given in Equation (20).

(20) $$s^{\alpha }\approx {\left(\frac{d}{b}\omega_{h}\right)}^{\alpha }\;\cdot \;\frac{ds^{2}+b\omega_{h}s}{d\left(1-\alpha \right)s^{2}+b\omega_{h}s+d\alpha }\prod_{k=-N}^{N}\frac{s+\omega_{z}}{s+\omega_{p}},\;\mathrm{for}\;0<\alpha <1$$

where *d* and *b* are the constants, and their values are set as 9 and 10 as recommended in [53]. Poles and zeros are determined as in Equation (21).

(21) $$\begin{array}{ccc} \omega_{p} & =\omega_{b}{\left(\frac{\omega_{h}}{\omega_{b}}\right)}^{\frac{k+N+\frac{1}{2}\left(1+\alpha \right)}{2N+1}}\;\;\omega_{z} & =\omega_{b}{\left(\frac{\omega_{h}}{\omega_{b}}\right)}^{\frac{k+N+\frac{1}{2}\left(1-\alpha \right)}{2N+1}} \end{array}$$

where $\omega_{h}$ and $\omega_{b}$ are the upper and lower limits of the frequency band, respectively. The lower and upper limits of the frequency band is defined as $10^{-4}\;\mathrm{rad}/s$ and $10^{4}\;\mathrm{rad}/s$, respectively.

The FOPID controller has increased its popularity in recent years. Fractional-order controllers are preferred because they perform better than traditional controllers, such as PID controllers [42]. However, designing the FOPID controller is not as simple as designing PID controllers because this controller includes two more parameters than the PID controller. The FOPID controller has five parameters, namely $K_{P}$, $K_{I}$, $K_{D}$, λ, and μ, where λ and μ, as non-integer real positive numbers, are the fractional powers of the integral and derivative operators. The transfer function of the FOPID controller is shown in Equation (22).

(22) $$G_{\mathrm{FOPID}}\left(s\right)=\frac{U(s)}{E(s)}=K_{P}+\frac{K_{I}}{s^{\lambda }}+K_{D}s^{\mu }$$

Cascade controllers are the controllers obtained by cascading different or similar controllers to each other. In this way, the degree of freedom value of the controller is increased, and better system responses can be obtained [31]. The FOPI-FOPD controller has six parameters, namely $K_{P1}$, $K_{P2}$, $K_{I}$, $K_{D}$, λ, and μ. The transfer function of the FOPI-FOPD controller is given in Equation (23).

(23) $$G_{\mathrm{FOPI}\text{-}\mathrm{FOPD}}\left(s\right)=\frac{U(s)}{E(s)}=\left(K_{P1}+\frac{K_{I}}{s^{\lambda }}\right)\left(K_{P2}+K_{D}s^{\mu }\right)$$

#### 2.5.3. Objective Function

Objective functions play a very important role in solving optimization problems. The objective functions commonly used in the literature for controller design are ITAE, IAE, ITSE, and ISE. Which objective function is preferred depends on the criteria of the optimization problem to be minimized or maximized. The IAE and ISE objective functions are insufficient to reduce the settling time. Conversely, the ITAE objective function minimizes the settling time. Furthermore, it directly affects the maximum overshoot [34,53]. The ITAE objective function is preferred to design the controller parameters because of the reasons given above. The mathematical expression of the ITAE is given in Equation (24).

(24) $$\mathrm{ITAE}=\int_{0}^{t_{\mathrm{sim}}}t\;\cdot \;\left(\left|∆f_{1}\right|+\left|∆f_{2}\right|+\left|∆P_{\mathrm{tie}}\right|\right)\;dt$$

where $∆f_{1}$ and $∆f_{2}$ are the frequency change of the system in the first and second areas, respectively. $∆P_{\mathrm{tie}}$ is the variation in the tie line power and $t_{\mathrm{sim}}$ is the simulation time.

## 3. Simulation Results and Discussion

### 3.1. Results of Mathematical Problems

The optimization results of the proposed FDB-AHA, AHA, and compared algorithms for the CEC 2017 and 2020 problems are obtained with 51 independent runs for each problem in 30, 50, and 100 dimensions. The maximum number of iterations is considered as 10,000 × Dimension. The study was performed in the MATLAB R2024a simulation environment. As a result of the statistical evaluations, the mean and standard deviation of the error values found for each problem because of 51 independent runs of the developed FDB-AHA and AHA on CEC 2017 and 2020 problems were calculated.

Table 3 shows the means and standard deviations of the error values for AHA and FDB-AHA on the CEC 2017 problems. This table shows the best result for each problem in bold. According to this, FDB-AHA scored better results than AHA in 25 out of 29 problems for 30 dimensions. From this table, the FDB-AHA shows better results than the AHA in all problems except four problems in 50 dimensions and one problem in 100 dimensions.

Table 4 shows the means and standard deviations of the error values for AHA and FDB-AHA on the CEC 2020 problems. It can be seen that FDB-AHA has a worse mean error value than AHA in only 1 out of 10 problems of 30 and 100 dimensions. For all CEC 2020 problems in 50 dimensions, FDB-AHA performs at least as well as AHA and provides better mean results for nine of the ten problems. In the 30-dimensional F8 (composition) problem and in the 100-dimensional F1 (unimodal) problem, FDB-AHA provides worse results than AHA. However, the fact that it generally has better averages than AHA in most problems is evidence that FDB-AHA has been successfully developed.

When Table 3 and Table 4 are considered together, it can be seen that FDB integration can limit performance on some problem landscapes. A significant proportion of the problems on CEC 2017 where FDB-AHA performed worse than AHA belong to the hybrid and composition classes. Similarly, in CEC 2020, the F8 problem, which exhibited lower performance for D=30, is also a composition problem. In such complex landscapes, taking distance into account in addition to the fitness value may, in some cases, reduce the algorithm’s exploitation capability by steering it away from promising local regions. However, it is also observed that AHA yields better results in some unimodal problems; in such structures where there is a strong convergence trend, the diversity provided by additional distance information may not always be advantageous. Therefore, whilst FDB improves overall performance, it may have a limiting effect in certain hybrid and composition problems, as well as in unimodal landscapes that require strong local convergence.

**Figure 5:**
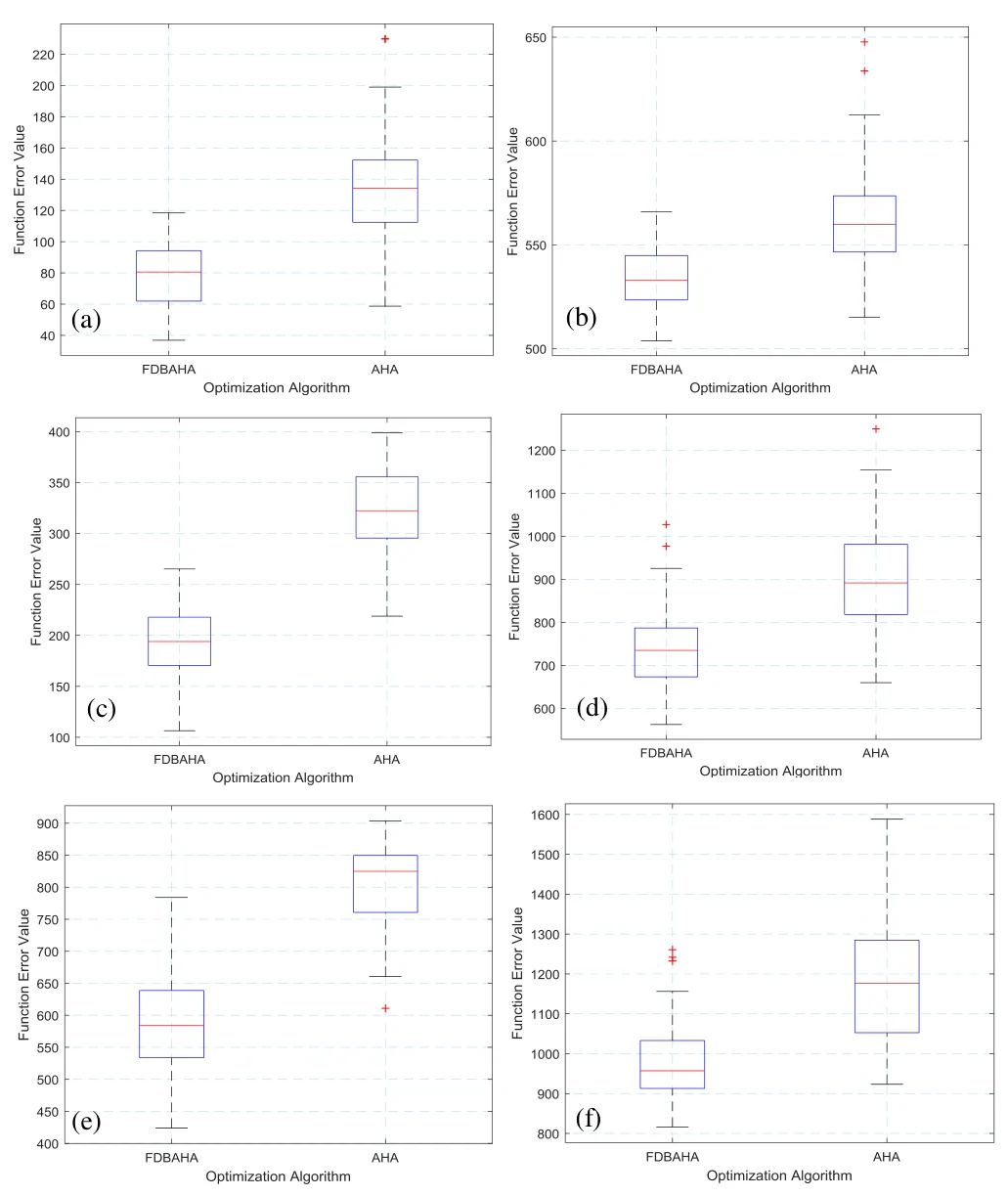
CEC 2017 box plots (blue boxes indicate the interquartile range, red lines indicate the median, and + symbols indicate outliers): (**a**) F5-D30, (**b**) F27-D30, (**c**) F5-D50, (**d**) F27-D50, (**e**) F5-D100, and (**f**) F27-D100.

The box-plot graphs of FDB-AHA and AHA for F5 and F27 problems in CEC 2017 with 30, 50, and 100 dimensions are shown in Figure 5. Figure 5a,c,e show the results of the F5 problem in 30, 50, and 100 dimensions, respectively. Meanwhile, Figure 5b,d,f show the results of the F27 problem in 30, 50, and 100 dimensions, respectively. It is seen from the figures that the developed FDB-AHA has a lower average than AHA in all dimensions for the considered problems. It is also observed from the box-plot graphs that the maximum and minimum value range of FDB-AHA is lower than AHA for all dimensions.

**Figure 6:**
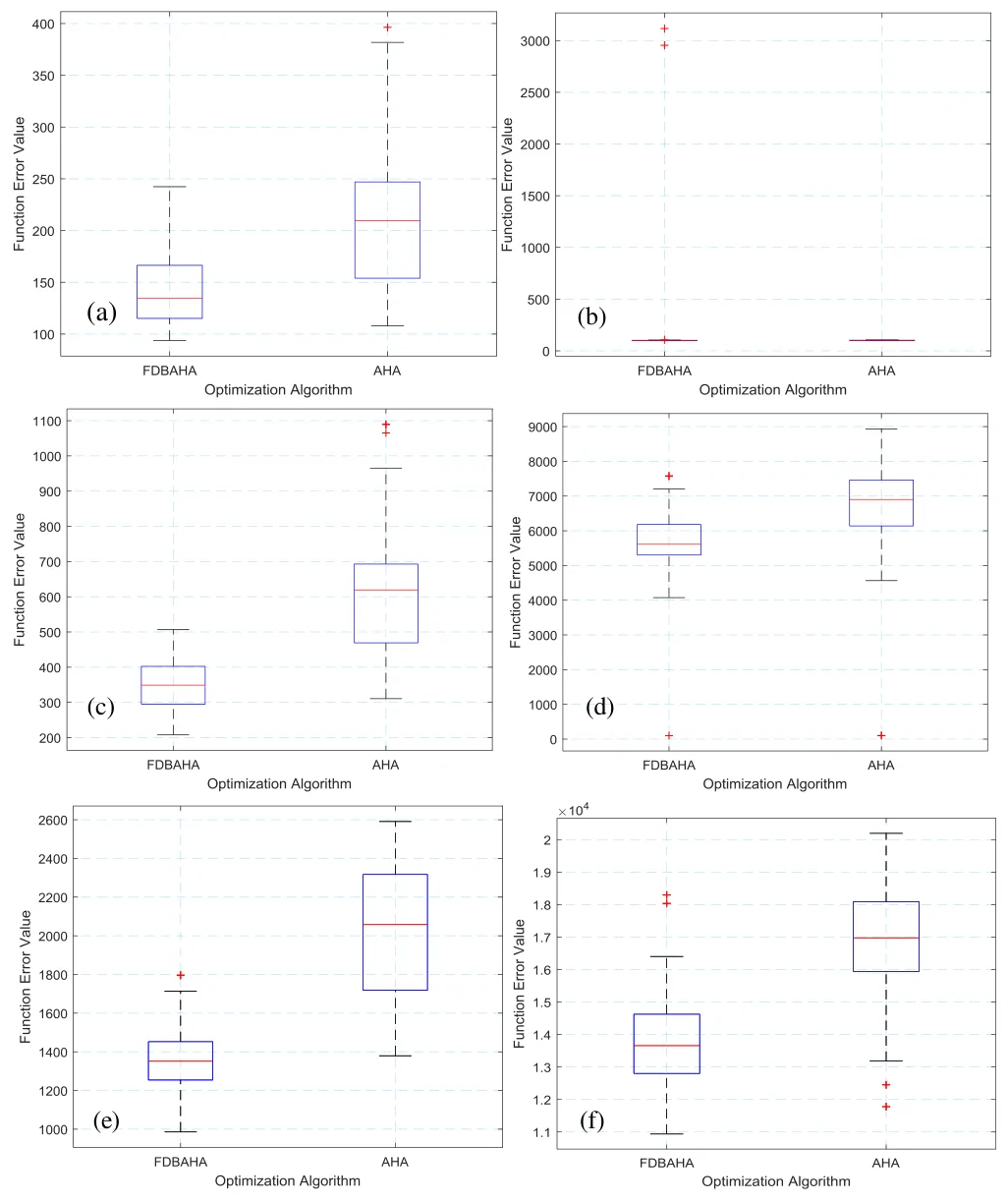
CEC 2020 box plots (blue boxes indicate the interquartile range, red lines indicate the median, and + symbols indicate outliers): (**a**) F3-D30, (**b**) F8-D30, (**c**) F3-D50, (**d**) F8-D50, (**e**) F3-D100, and (**f**) F8-D100.

The box-plot graphs of FDB-AHA and AHA for the functions F3 and F8 in the list of CEC 2020 with 30, 50, and 100 dimensions are shown in Figure 6. Figure 6a,c,e show the results of the F3 problem in 30, 50, and 100 dimensions, respectively. Meanwhile, Figure 6b,d,f show the results of the F8 problem in 30, 50, and 100 dimensions, respectively. From these plots, it is seen that the developed FDB-AHA generally provides better results than AHA.

Wilcoxon and Friedman tests are comparison tests used in non-parametric data analysis with non-normal distribution. Friedman tests are used to determine the statistical difference between groups in a dataset. In this study, the benchmark functions optimized by each algorithm can be considered as groups. The Friedman test determines whether there is a statistically significant difference between these groups. This difference allows us to see how each group performs better or worse than the other groups. For this purpose, the Wilcoxon test is used to compare the performance of any two groups with each other using the differences between the groups [54]. The performance of the developed FDB-AHA is compared with some successful algorithms selected from the literature. The results of FDB-AHA and the competing algorithms for CEC 2017 and 2020 problems are obtained for 30, 50, and 100 dimensions, equal iterations and 51 independent runs and Friedman and Wilcoxon analyses are applied. Table 5 shows the mean ranking values of FDB-AHA and the competitor algorithms according to Friedman analysis. In the table, the ranking values of the leading algorithm are bold. Therefore, while the improved FDB-AHA algorithm ranked first across all dimensions in the CEC 2017 problems, it ranked second in one dimension in the CEC 2020 problems. In CEC 2020 problems, it is observed that the PO algorithm is first and FDB-AHA is second in 50 dimensions. In addition, the fact that the AHA is 9th out of 12 algorithms according to the mean rank values and the FDB-AHA version is the leader, leaving 8 algorithms behind, that reveals the success of the development work.

Table 6 shows the pairwise comparison results obtained in the Wilcoxon analysis of FDB-AHA and competing algorithms. In the table, the results in which the FDB-AHA is superior are bolded. According to these results, FDB-AHA is superior to all algorithms except the PO algorithm. Between PO and FDB-AHA, there is only a small difference. For example, for CEC 2020 problems, a draw was observed between FDB-AHA and PO with three wins, four draws, and three defeats in 30 dimensions, four wins, two draws, and four defeats in 50 dimensions, and three wins, dour draws, and three defeats in 100 dimensions. In CEC 2017 problems, in all dimensions, the number of problems in which the PO algorithm wins over FDB-AHA is one more than the number of problems in which it loses.

**Figure 7:**
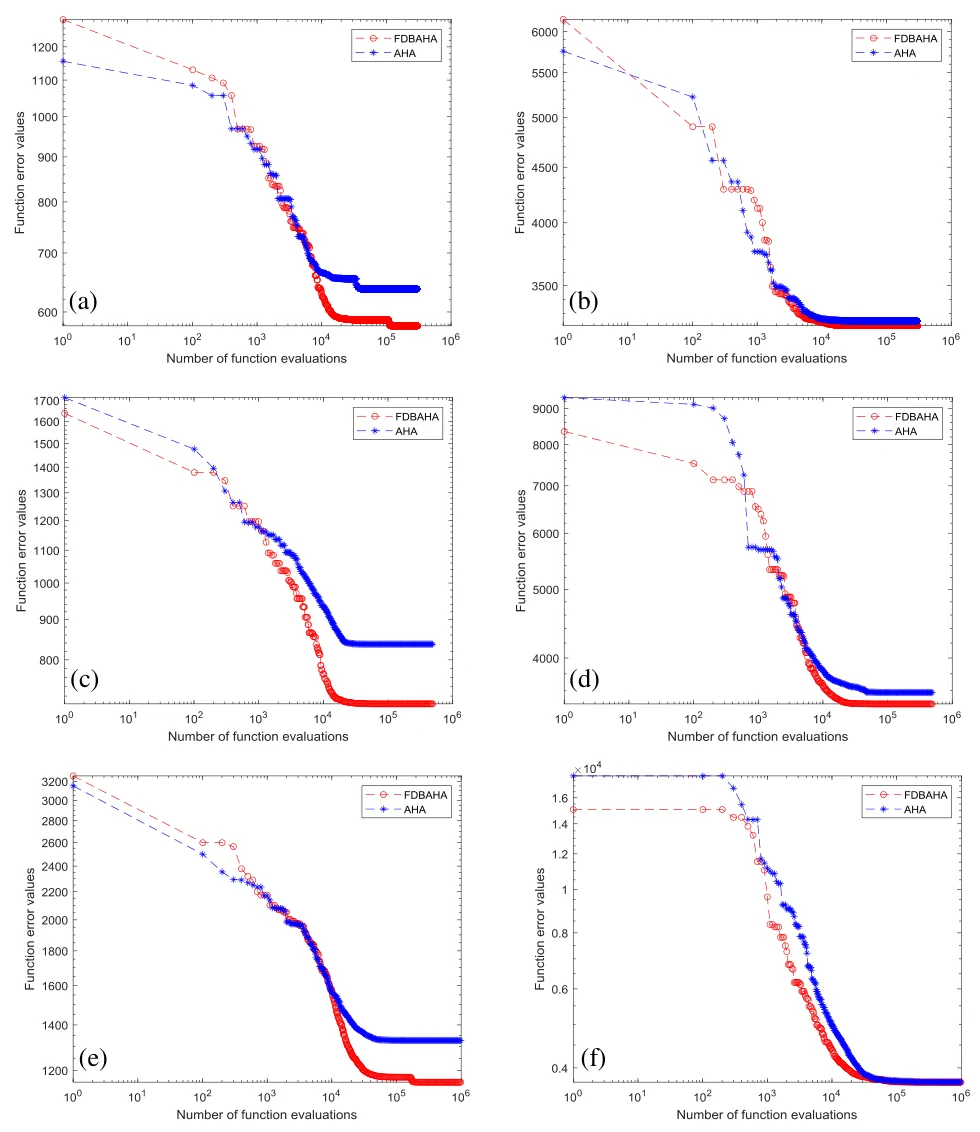
CEC 2017 convergence curves: (**a**) F5-D30, (**b**) F27-D30, (**c**) F5-D50, (**d**) F27-D50, (**e**) F5-D100, and (**f**) F27-D100.

**Figure 8:**
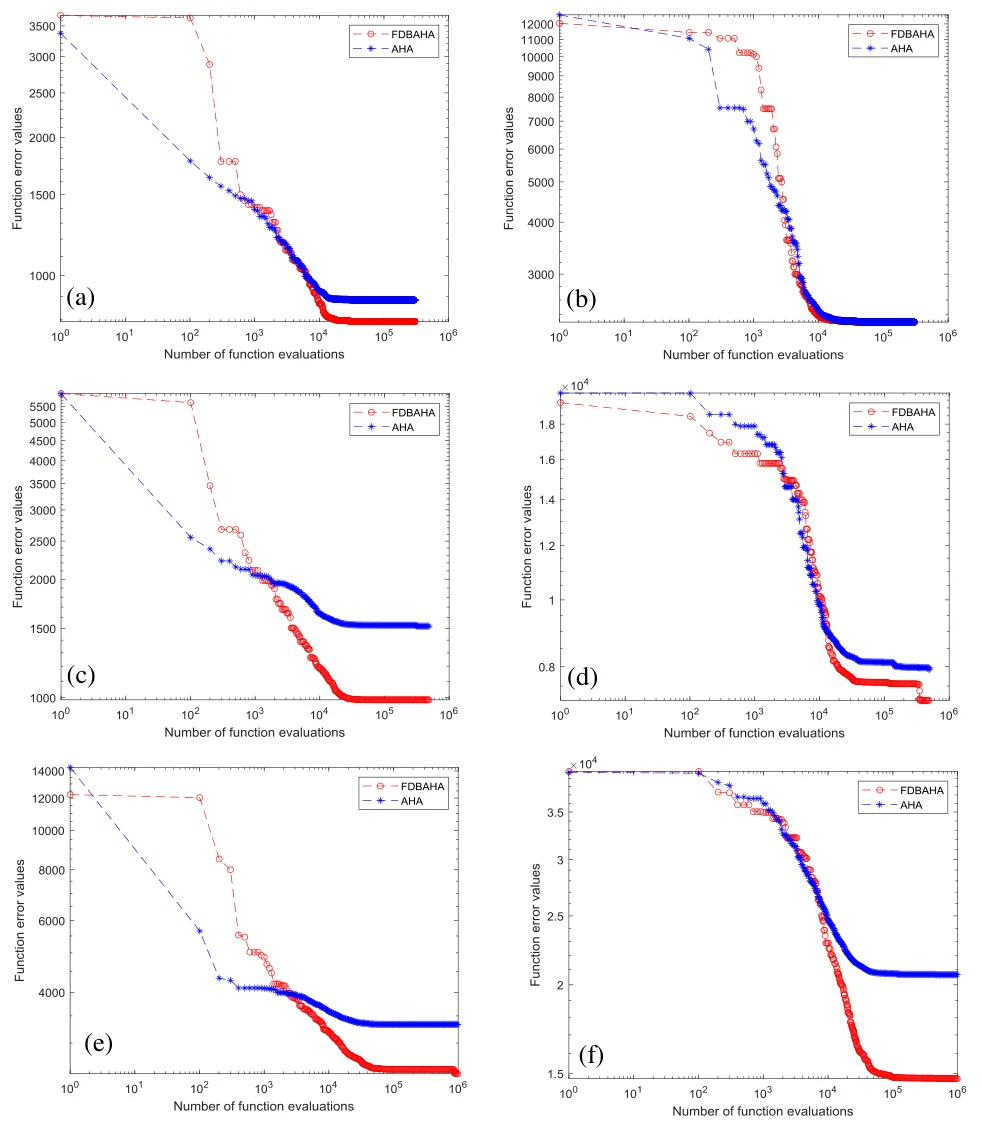
CEC 2020 convergence curves: (**a**) F3-D30, (**b**) F8-D30, (**c**) F3-D50, (**d**) F8-D50, (**e**) F3-D100, and (**f**) F8-D100.

The convergence curve is a graph showing the change in the objective function value with respect to iteration during the optimization process. At the end of each iteration, a new objective function value is found, and the best solution is determined after a set number of iterations. With the convergence curve, it can be observed how long this process takes and how successful it is. The convergence curve can also be used to determine whether the time allocated for optimization is sufficient. The convergence curves of FDB-AHA and AHA used in solving the problems are depicted in Figure 7 for CEC 2017. Figure 7a,c,e show the convergence curves of the F5 problem in 30, 50, and 100 dimensions, respectively. Meanwhile, Figure 7b,d,f show the convergence curves of the F27 problem in 30, 50, and 100 dimensions, respectively. For CEC 2020, the convergence curves are presented in Figure 8. Figure 8a,c,e show the convergence curves for F3, while Figure 8b,d,f show the convergence curves for F8. It is observed that the developed FDB-AHA converges earlier and better than the basic AHA for most problems and in all dimensions.

As a final analysis, the runtime of AHA and FDB-AHA were compared to evaluate the computational costs of the algorithms. Considering that run times may vary depending on hardware specifications, the operating environment, and the software version, the measurements were performed in the MATLAB R2024a environment on a computer equipped with an Intel^®^ Core™ i7-8750H CPU @ 2.20 GHz and 16 GB of RAM. In a single independent run for D=100, the runtime of AHA in the CEC 2017 tests was measured at 345.65 s, while that of FDB-AHA was 483.92 s. In the CEC 2020 tests, these runtimes were 659.44 s and 796.70 s, respectively. The results show that FDB-AHA has a longer runtime compared to the standard AHA because the FDB-based candidate selection mechanism requires additional computations. However, when the improvement in solution quality and the additional computational cost are considered together, the performance gain provided by FDB-AHA is found to be significant. Furthermore, since the controller parameters in LFC applications can be optimized offline once and then reused, the impact of the additional computation time during the optimization phase on practical applications remains limited.

### 3.2. Results of the Power System Problems

#### 3.2.1. Two-Area Non-Reheat Thermal/Thermal System (System 1)

The parameter values of PID, FOPID, and FOPI-FOPD controllers for LFC in System 1 are designed with FDB-AHA, and the optimal controller parameters with the best objective function value obtained from 10 independent runs are given in Table 7. The controller parameters are determined by applying a 10% step load to the first area at t=0s. In the studied system, the parameter values of both areas are assumed to be equal to each other. Furthermore, the controllers designed for both areas have the same parameter values. In this power system, the constraints of the controller parameters are selected as $\left[K_{P},K_{I},K_{D}\right]\in \left[0,3\right]$ for the PID, $\left[K_{P},K_{I},K_{D}\right]\in \left[0,3\right]$ and [λ,μ]∈[0,1.5] for the FOPID, and finally, $\left[K_{P1},K_{P2},K_{I},K_{D}\right]\in \left[0,3\right]$ and [λ,μ]∈[0,1.5] for the FOPI–FOPD controllers. As shown in Table 7, in System 1, the approach to the bound value occurs particularly in the integral gain. For example, in the FOPID controller, $K_{I}=2.9999$, and in the FOPI-FOPD controller, $K_{P2}=2.9999$ and $K_{I}=2.9999$ reach the upper bound. This indicates that, in the optimal design, increasing the integral gain is particularly preferred.

**Figure 9:**
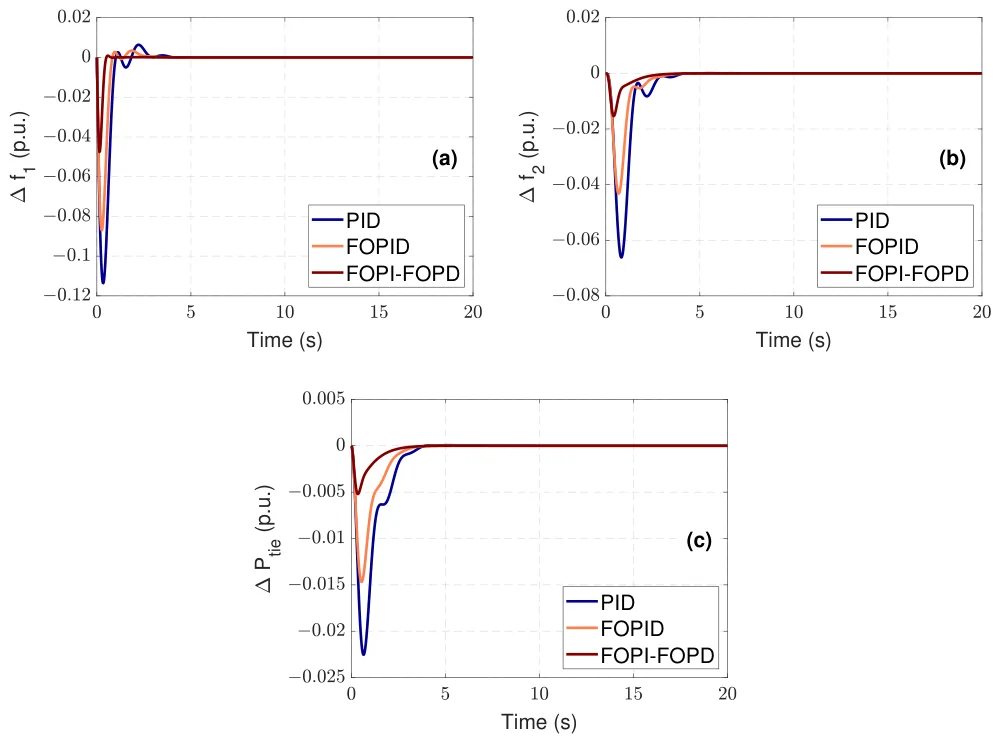
Controller responses for System 1: (**a**) $∆f_{1}$, (**b**) $∆f_{2}$, and (**c**) $∆P_{\mathrm{tie}}$.

In Table 8, minimum objective function values and settling time, as one of the important time-domain specifications, of the controllers are given with the results of some existing studies in the literature. Among the considered controllers, the FDB-AHA-based FOPI-FOPD controller yields the lowest objective function value together with the shortest settling times for the frequency deviations in both areas, $∆f_{1}$ and $∆f_{2}$, and the tie-line power deviation, $∆P_{\mathrm{tie}}$. Furthermore, to compare the performances of the designed controllers, time-domain responses of the controllers are shown in Figure 9. Figure 9a–c show $∆f_{1}$, $∆f_{2}$, and $∆P_{\mathrm{tie}}$, respectively.

**Figure 10:**
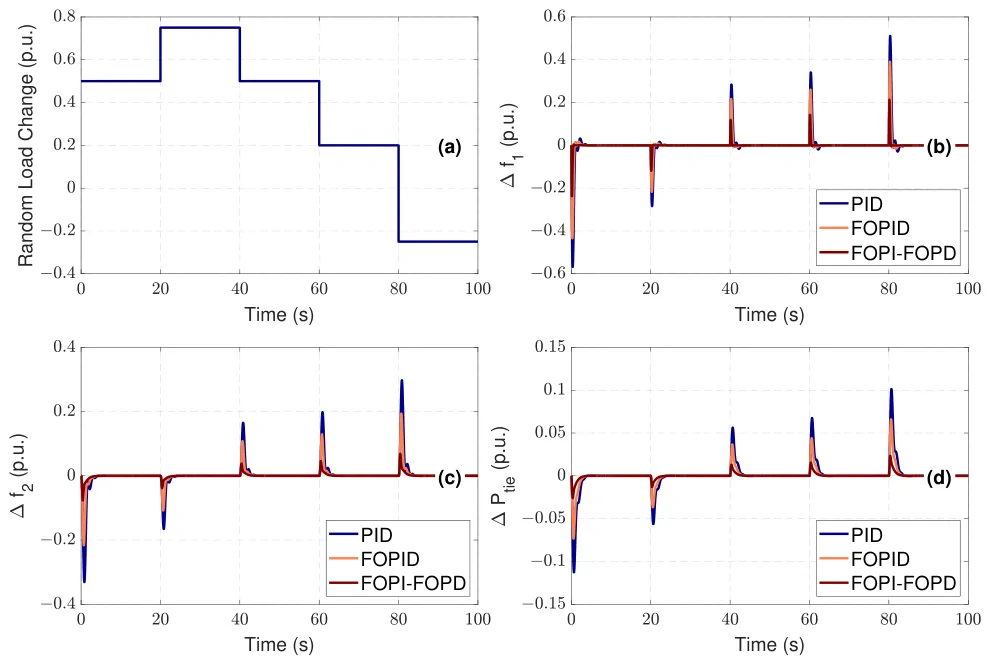
System 1 responses under load variation: (**a**) load signal, (**b**) $∆f_{1}$, (**c**) $∆f_{2}$, and (**d**) $∆P_{\mathrm{tie}}$.

The stability and reliability of the controllers designed with the developed FDB-AHA for LFC in System 1 are tested by disturbance load analysis. In this context, the disturbance load signal shown in Figure 10a is applied to the system and the system responses are observed. The changes in the system frequency in the first and second area and tie-line power variation because of the applied disturbance load at different rates are shown in Figure 10b–d for each controller, respectively. It is observed from this analysis that the PID, FOPID, and FOPI-FOPD controllers designed with the developed FDB-AHA maintain the system stability against different values of applied disturbance load and the FDB-AHA performs a successful optimization process. Furthermore, the figure demonstrates that the FOPI-FOPD controller exhibits reduced overshoot and undershoot, as well as a shorter settling time, in comparison to both the PID and FOPID controllers.

The frequency and tie-line power variation are affected by the sudden load variation in the system as well as the change in system dynamics. Therefore, the designed controller should have sufficient robustness against external factors affecting system stability and variations in system parameters. Robustness analysis is performed to measure the robustness of the FOPI-FOPD controller, which shows the best performance among the controllers designed for System-1, in case of parameter changes in the system. During the analysis, the $T_{g}$, $T_{t}$, $T_{12}$, B, and R parameter values of the system are varied by ±25% and ±50%. Based on this, the settling time and undershoot values of the system frequency and tie-line power variation are shown in Table 9 for the FOPI-FOPD controller. In general, while alterations to any parameter value may impact performance, the system remains stable.

**Figure 11:**
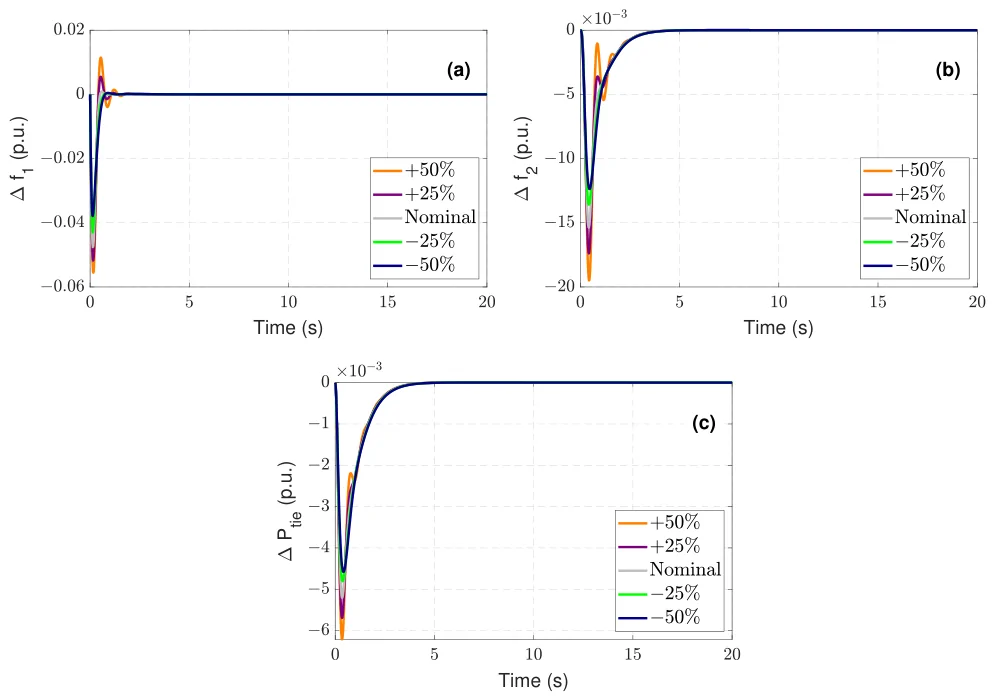
System 1 FOPI-FOPD responses under $T_{g}$ variation: (**a**) $∆f_{1}$, (**b**) $∆f_{2}$, and (**c**) $∆P_{\mathrm{tie}}$.

Figure 11a–c show $∆f_{1}$, $∆f_{2}$, and $∆P_{\mathrm{tie}}$ for the perturbation in the $T_{g}$ parameter, respectively. It is evident that the controllers designed with the FDB-AHA effectively maintain system stability in the face of variations in system parameters.

#### 3.2.2. Two-Area Multi Source Power System (System 2)

The parameter values of PID, FOPID, and FOPI-FOPD controllers for LFC in System 2 are designed with FDB-AHA, and the optimal controller parameters with the best objective function value obtained from 10 independent runs are given in Table 10. The controller parameters are determined by applying a 1% step load to the first area at t=0s. The same controllers with different parameters are designed for each source and the two areas are considered as equal to each other in the system. The constraints of the controller parameters are selected as $\left[K_{P},K_{I},K_{D}\right]\in \left[-2,2\right]$ for the PID, $\left[K_{P},K_{I},K_{D}\right]\in \left[-2,2\right]$ and [λ,μ]∈[0,1.5] for the FOPID, and finally, $\left[K_{P1},K_{P2},K_{I},K_{D}\right]\in \left[-2,2\right]$ and [λ,μ]∈[0,1.5] for the FOPI-FOPD controllers. The results in Table 10 show that the limit effect manifests in different controller components across various generation sources. For example, in the PID structure, the value $K_{P}=1.9999$ for thermal and hydro sources indicates that the proportional gain is dominant, whereas for the gas source, the values $K_{I}=1.9999$ and $K_{D}=1.9898$ indicate that the integral and derivative gains are more pronounced.

**Figure 12:**
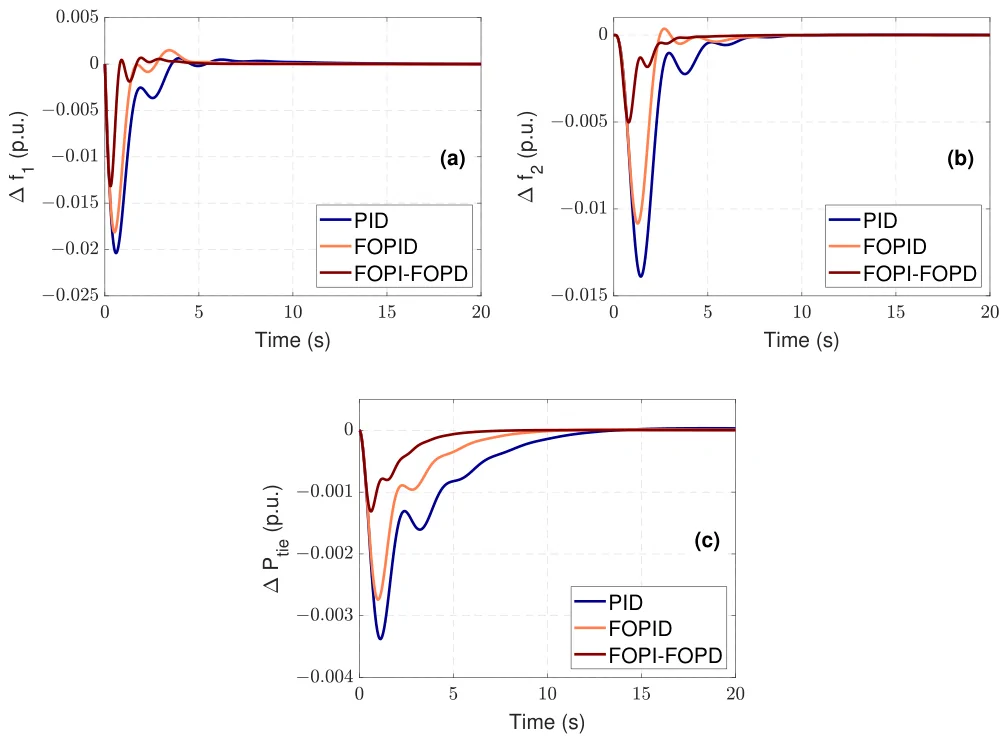
Controller responses for System 2: (**a**) $∆f_{1}$, (**b**) $∆f_{2}$, and (**c**) $∆P_{\mathrm{tie}}$.

ITAE values and the settling times are presented in Table 11. Furthermore, time-domain responses of $∆f_{1}$, $∆f_{2}$, and $∆P_{\mathrm{tie}}$ for each controller are shown in Figure 12a–c, respectively. It is observed that FDB-AHA-based FOPI-FOPD is the controller with the best objective function and settling time among the controllers for System-2. In addition, if recent studies are considered, it can be said that the performance of the PID controller designed with FDB-AHA performs better than the PID controllers designed with other MHS algorithms.

**Figure 13:**
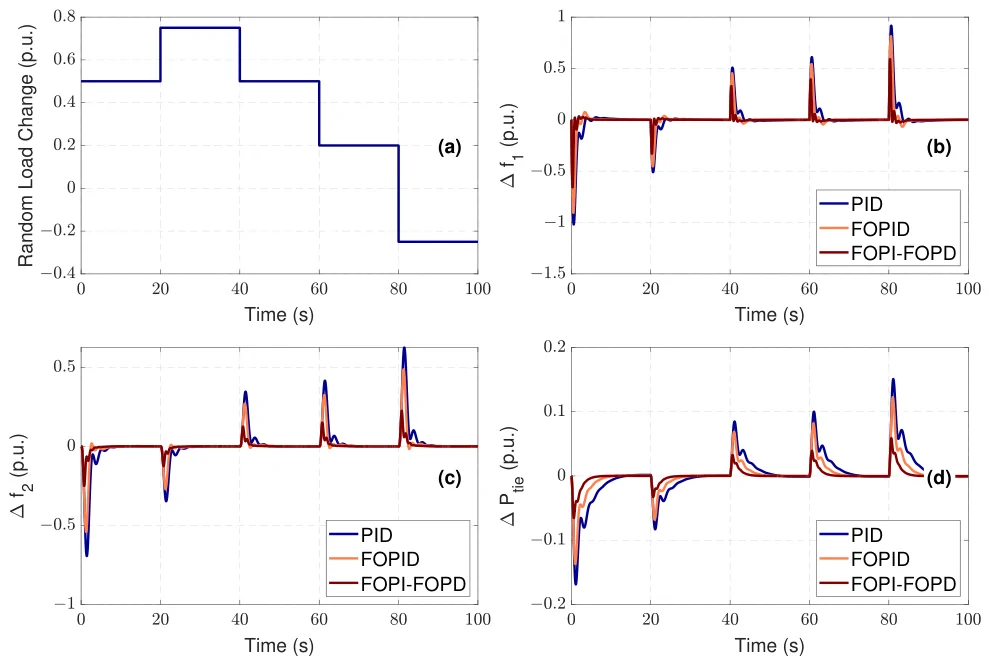
System 2 responses under load variation: (**a**) load signal, (**b**) $∆f_{1}$, (**c**) $∆f_{2}$, and (**d**) $∆P_{\mathrm{tie}}$.

To test the stability and reliability of the proposed controllers designed with the developed FDB-AHA for System 2, a disturbance load analysis is performed by applying a load signal to the first area of the system as shown in Figure 13a. The $∆f_{1}$, $∆f_{2}$, and $∆P_{\mathrm{tie}}$ changes for each controller are shown in Figure 13b–d, respectively. It is obvious that the controllers exhibit stable performance against random load variations and successfully ensure system stability.

**Figure 14:**
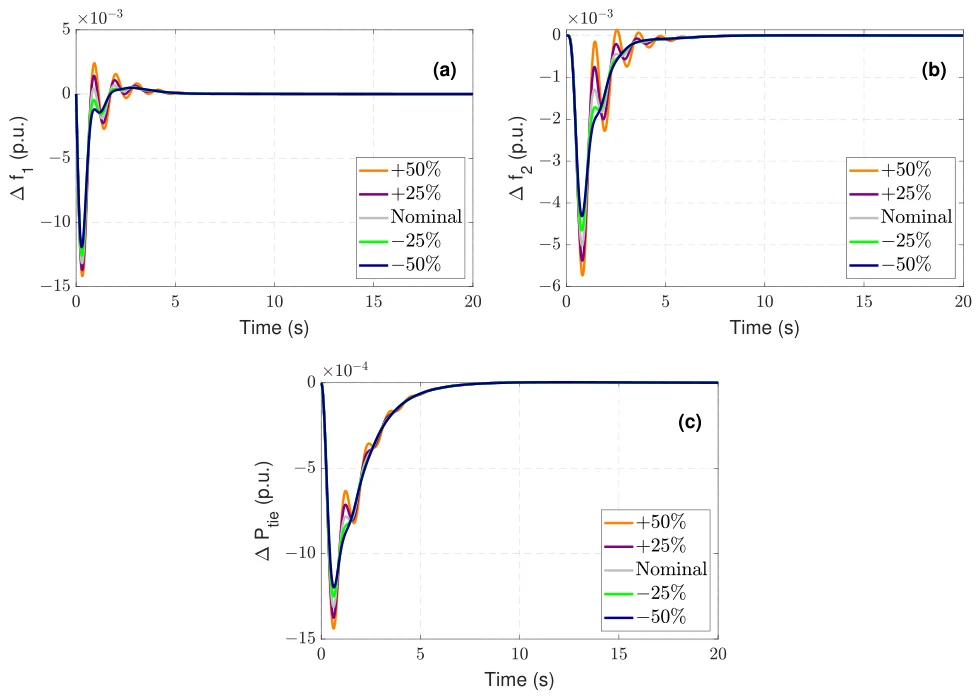
System 2 FOPI-FOPD responses under $T_{g}$ variation: (**a**) $∆f_{1}$, (**b**) $∆f_{2}$, and (**c**) $∆P_{\mathrm{tie}}$.

The performance of the FOPI-FOPD controller designed with the FDB-AHA for System 2 is tested with robustness analysis and the results are given in Table 12. The robustness of the controller is tested by varying the parameter values of $T_{g}$, $T_{t}$, $T_{12}$, *B*, and *R* by ±25% and ±50%. Figure 14a–c show $∆f_{1}$, $∆f_{2}$, and $∆P_{\mathrm{tie}}$ for the perturbation in the $T_{g}$ parameter, respectively. When the robustness analysis results for System 2 are analyzed, it can be concluded that the developed FDB-AHA is very effective in controller design. Although the designed controller is slightly affected by varying the system parameters, it maintained its stability and provided a successful LFC solution.

#### 3.2.3. Two-Area PV/Thermal Power System (System 3)

The optimum controller parameters of PID, FOPID, and FOPI-FOPD controllers for the LFC in System 3 is designed with the improved FDB-AHA, and the optimized controller parameters with the best objective function value obtained from 10 independent trials are given in Table 13. The controllers are designed by applying a 10% step load to the second area at t=0s. The controllers designed for both areas have different parameter values. In System 3, the constraints of the controller parameters are selected as $\left[K_{P},K_{I},K_{D}\right]\in \left[0,2\right]$ for the PID, $\left[K_{P},K_{I},K_{D}\right]\in \left[0,2\right]$ and [λ,μ]∈[0,1.5] for the FOPID, and finally, $\left[K_{P1},K_{P2},K_{I},K_{D}\right]\in \left[0,2\right]$ and [λ,μ]∈[0,1.5] for the FOPI-FOPD controllers. Table 13 shows that thermal source controllers, in particular, approach their bound values. For the PID controller, the values $K_{P}=1.9999$ and $K_{I}=1.9999$ were obtained, while for the FOPI-FOPD controller, the values $K_{P1}=1.9999$, $K_{P2}=1.9999$, and $K_{I}=2$ were obtained. These results indicate that proportional and integral effects are more dominant in the optimal design for the thermal source.

**Figure 15:**
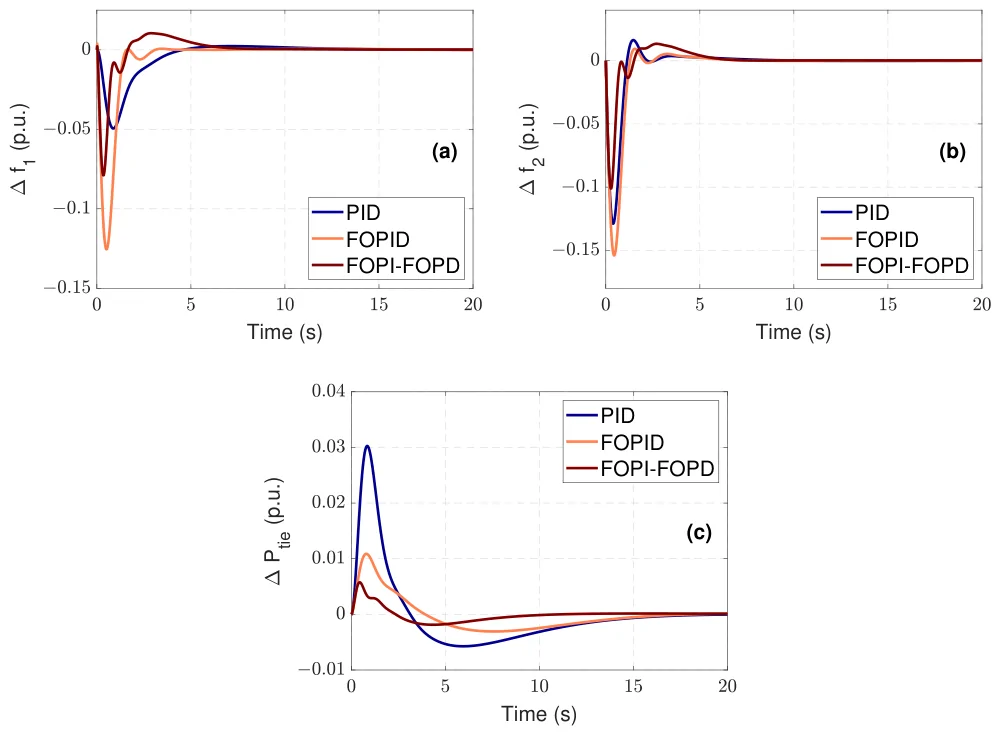
Controller responses for System 3: (**a**) $∆f_{1}$, (**b**) $∆f_{2}$, and (**c**) $∆P_{\mathrm{tie}}$.

Three different controllers are designed with the proposed FDB-AHA for System 3 and their performances are observed through time-domain analysis. The ITAE values and the settling times of $∆f_{1}$, $∆f_{2}$, and $∆P_{\mathrm{tie}}$ for each controller are given in Table 14. In addition, the results of some existing studies are added to the table to compare the performance of the proposed controllers. Table 14 shows that the FOPI-FOPD controller designed using FDB-AHA has the lowest ITAE value. However, when the settling times are analyzed, especially for $∆f_{1}$, the best result is obtained with the FOPID controller. Figure 15a–c show $∆f_{1}$, $∆f_{2}$, and $∆P_{\mathrm{tie}}$ in System 3, respectively. As can be seen in Figure 15c, the FOPI-FOPD controller has by far the best performance in eliminating the tie-line power variation.

**Figure 16:**
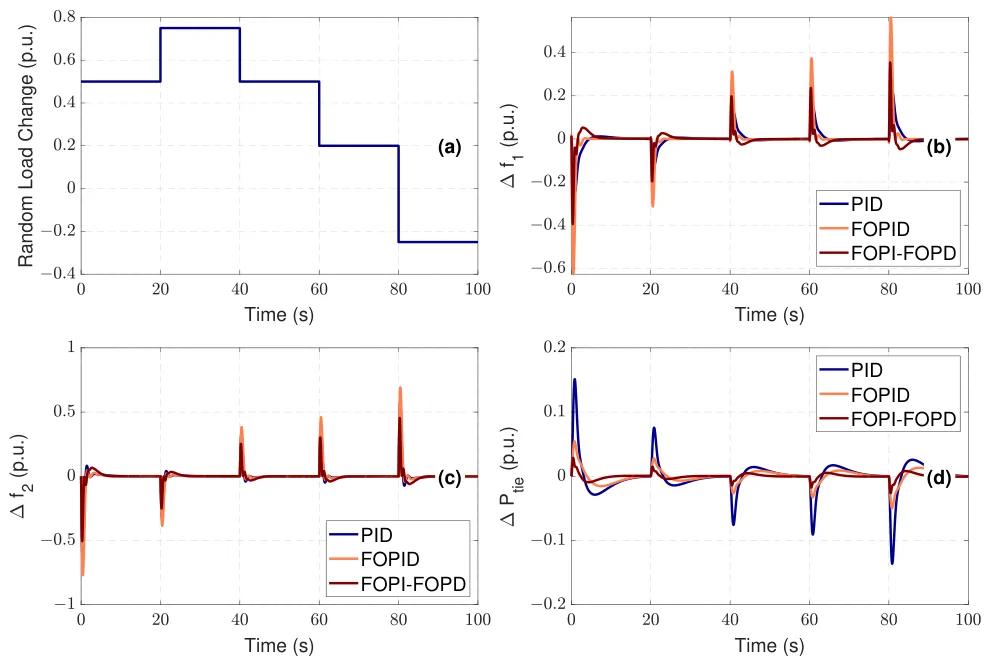
System 3 responses under load variation: (**a**) load signal, (**b**) $∆f_{1}$, (**c**) $∆f_{2}$, and (**d**) $∆P_{\mathrm{tie}}$.

Disturbance load analysis is once again carried out for System 3 as with Systems 1 and 2. For this, a load variation signal, shown in Figure 16a, is applied to the second area and frequency changes in the areas and tie-line power variation are obtained as in Figure 16b–d.

**Figure 17:**
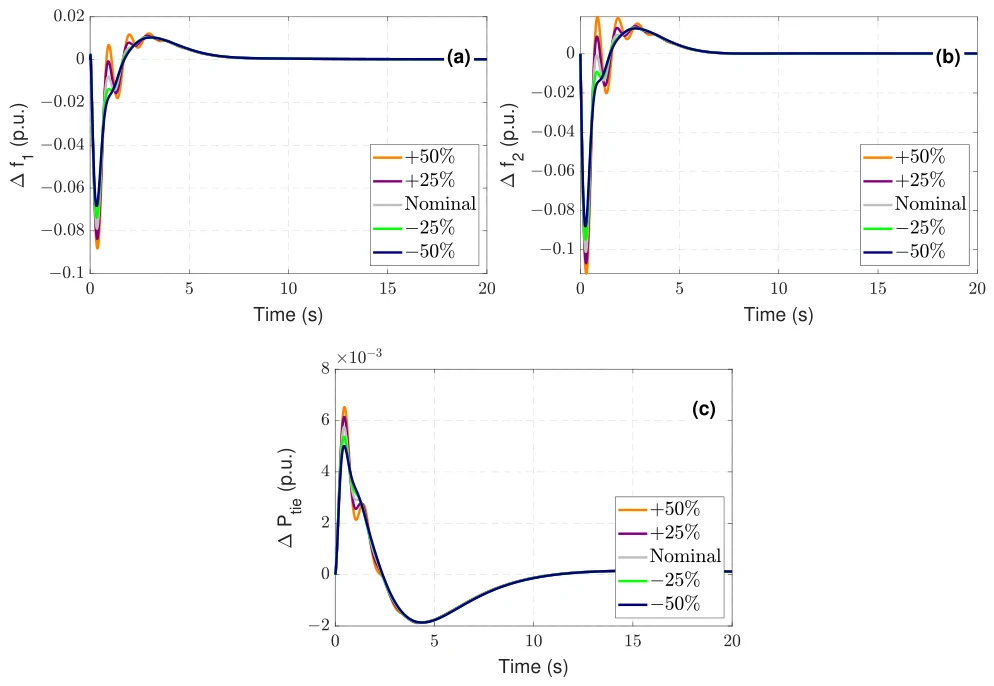
System 3 FOPI-FOPD responses under $T_{g}$ variation: (**a**) $∆f_{1}$, (**b**) $∆f_{2}$, and (**c**) $∆P_{\mathrm{tie}}$.

The performance of the FOPI-FOPD controller designed with FDB-AHA for System-3 is tested using robustness analysis and the results are given in Table 15. The robustness of the controller is tested by varying the parameter values of $T_{g}$, $T_{t}$, $T_{12}$, *B*, and *R* of the thermal source of the system by ±25% and ±50%. Moreover, to better observe the performance of the designed FOPI-FOPD controller with respect to the varying of the $T_{g}$ system parameter, $∆f_{1}$, $∆f_{2}$, and $∆P_{\mathrm{tie}}$ are depicted in Figure 17a–c, respectively. As a result of the robustness analysis, It can be posited that the FOPI-FOPD controller designed with FDB-AHA is robust against parameter uncertainty in System 3 and performs well in ensuring system stability.

#### 3.2.4. Validation via Real-Time Simulator

**Figure 18:**
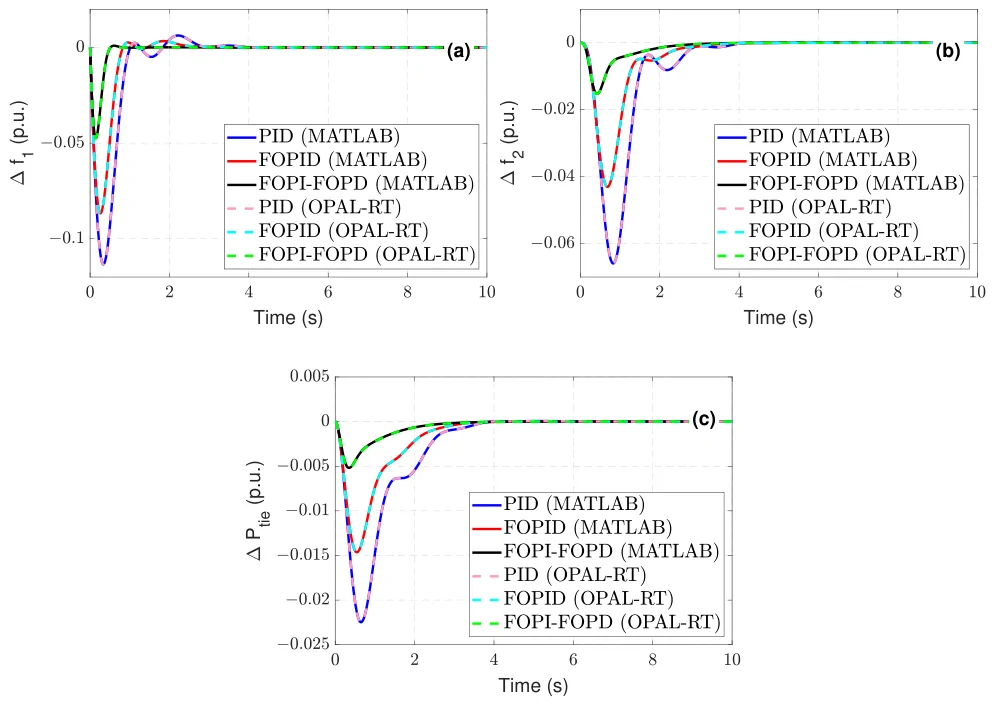
MATLAB and OPAL-RT responses: (**a**) $∆f_{1}$, (**b**) $∆f_{2}$, and (**c**) $∆P_{\mathrm{tie}}$.

To evaluate the agreement between the OPAL-RT real-time simulation results and the offline MATLAB results using the same sampling time of 0.01 s, the frequency deviations and tie-line power changes for System 1 were compared. The $∆f_{1}$, $∆f_{2}$, and $∆P_{\mathrm{tie}}$ responses obtained in the MATLAB and OPAL-RT environments are shown in Figure 18. As seen in the figure, the real-time and offline responses match to a large extent. To quantitatively evaluate this agreement, the Mean Squared Error (MSE) values between the MATLAB and OPAL-RT signals were also calculated, and these are presented in Table 16. It can be seen that the MSE values for all controller and output variables are very low. The highest MSE value was obtained for the $∆f_{1}$ response of the FOPI-FOPD controller, at $1.83\times 10^{-9}$.

## 4. Conclusions

AHA is a metaheuristic search method that models the foraging behavior of hummingbirds. The algorithm design utilizes the flight techniques, foraging strategies, and memory of hummingbirds. When the vanilla AHA algorithm was tested, it was concluded that there were deficiencies in the exploration capability and that there was not a appropriate balance between exploration and exploitation. To overcome these deficiencies, the FDB method was integrated into the foraging strategies of the AHA by fine-tuning. The FDB-AHA obtained because of this integration process was compared with AHA and 10 other well-known MHS algorithms in the literature. The comparison was performed using Friedman and Wilcoxon statistical test methods on −30-, −50-, and −100-dimensional CEC 2017 and 2020 problems. The results obtained from the Friedman statistical test method show that FDB-AHA has the best result among the 12 algorithms, while AHA ranks 9th. In addition, a pairwise comparison of FDB-AHA with the other 11 algorithms using the Wilcoxon statistical test method shows that FDB-AHA performs better as the problem complexity increases. These statistical test results prove that the performance of AHA has ben significantly improved by the addition of FDB. Additionally, the numerical results were reinforced by convergence figures and box-plot graphics, which illustrated the convergence speed, performance, and maximum, minimum, and mean values achieved by the proposed algorithm.

The improved FDB-AHA is additionally tested in LFC, which is considered an important problem in power-system and control-system engineering. In this context, three different power systems, namely Thermal/Thermal, Thermal/Hydro/Gas, and PV/Thermal, and three different controller structures (PID, FOPID, FOPI-FOPD) are considered. The controller parameters are determined by minimizing the ITAE objective function. The optimal results obtained are compared with the results of studies in the literature using the same power systems in terms of time-domain analysis, and the superior performance of the proposed FDB-AHA is proved. In addition, the performance of the controllers under robustness and changing load is also tested for the obtained optimum results and it is proved that the results show a stable controller design. Finally, the results obtained are verified by testing the controllers designed using FDB-AHA in a real-time simulator (OPAL-RT).

## Figures and Tables

**Table 1:** Summary of MHS algorithm improvement studies.

Ref.	Type	Alg.	Modification	Imp. Alg.	Problem
[5]	D	BO	Self-adaptive parameters based on repulsion-based learning	SA-BO	CEC2014 and Powered Knee Orthotic Device
[6]	D	CS	Adaptive parameter control based on Cauchy distribution and Lehmer mean	A-CS	CEC2013, fractional-order financial system, and Rössler system
[7]	D	PPE	Chaotic maps applied to the initial population	C-PPE	CEC2013 and stock prediction
[8]	D	SC	Singer Map used to generate initial solution candidates	C-SC	Hadith and UCI datasets
[9]	D	ACO	Normal distribution replaced by Levy flight	L-ACO	TSPLIB
[10]	D/H	BH	Golden Sine (GS) operators and Levy flight mechanism	L-GS-BH	CEC2005 and CEC2017
[11]	H	ACO, BSO	ACO pheromone-based learning combined with BSO dynamic position updating	ACO-BSO	Cloud computing load balancing
[12]	H	AO, ArO	Equal-probability solution updating using AO and ArO	AO-ArO	Classical benchmark problems
[13]	H	GWO, HHO	GWO exploitation combined with HHO exploration	GWO-HHO	UCI feature-selection datasets
[14]	H	HHO, MFO	HHO exploitation, MFO exploration, chaotic maps, and EP dynamics	EP-C-HHO-MFO	CEC2005 and engineering problems
[15]	H	PSO, SS	Initial population generated by PSO followed by search using SS	PSO-SS	Software defect datasets
[16]	H	SA, MRFO	Initial population generated by SA followed by search using MRFO	SA-MRFO	AVR controller design
[18]	S	E	Level-based Population Model (LPM) and RW selection	LPM-RW-E	Feature selection
[19]	S	TS	Random tree selection replaced by RW	RW-TS	Classical benchmark problems
[20]	S	AnO	Tournament selection integrated into the random walk stage	T-AnO	Benchmark problems and QAPLIB
[21]	S	FO	Roulette wheel selection replaced by tournament selection	T-FO	Classical benchmark problems
[22]	S	ACO	Epsilon-Greedy (EG) selection and Levy flight	EG-L-ACO	TSPLIB
[23]	S	B	Greedy Crossover (GC) used for solution generation	GC-B	ALL-AML, CNS, and other feature-selection datasets
[24]	S	AEO	FDB integrated into the decomposition stage and RW used for candidate selection	RW-FDB-AEO	Welded Beam and Rolling Element Bearing problems
[25]	S	AG-DE	FDB integrated into the mutation stage and RW used for candidate selection	RW-FDB-AG-DE	CEC2014, CEC2017, and IEEE 30-bus SCOPF
[26]	S	WChOA	FDB integrated into the guidance mechanism	FDB-WChOA	CEC2020 and multilevel thresholding color image segmentation
[27]	S	SFOA	FDB/dFDB-based guide selection integrated into the search process	FDBSFOA, dFDBSFOA	CEC2017, CEC2020, CEC2022, and constrained engineering design problems

D: Dynamic parameter tuning; H: Hybrid design; S: Selection method; B: Bat, E: Evolutionary Alg.: Algorithm; Imp. Alg.: Improved algorithm.

**Table 2:** Summary of LFC studies.

Ref. No	Controller Type	Algorithm	Obj. Func.	Source	Area
[29]	PI/PID	BWO	ITAE	PV-T	2
[30]	PID/TID	GWO-CS	ITAE	PV-T	2
[31]	FOPI-FOPD	DS	ITAE	T-H-G	1-2-3
[32]	MFOPID	JSO	ITSE	T-H-G-RES	2
[33]	FOPI	LA	ISE	T-H-G	2
[34]	PID/Fuzzy-PI/Fuzzy PID	HS-CS	ITAE	T-H-G	2
[35]	PI/PID/2DOF-PID	TLBO	ITAE	T-H-G	2
[36]	PID/Fuzzy-PID	I-ACO	$I_{All}$	T-H	2
[37]	PID/Fuzzy-PID	LUS-TLBO	ITAE	T-H-G	2
[38]	PID/PI-PD	I-GWO	ITAE	T-H-G-EV	1-2-5
[39]	PI/PID/PI-PD	SFS-PS	ITAE	T-H-G-EV	1-2-3
[40]	PI/PID	SFL-PS	ITAE	PV-T	2
[41]	PID/PIDF	I-WOA	ITAE	PV-T	2
[42]	FOPID	CIO	ITAE	T	1-2
[43]	PID/PI-PD/TID/FOPI-FOPID	L-IC	ITAE	T-H	2
[44]	PID/TIDF	GA/PSO/DE	ITAE	T	2-3
[45]	I/PI/PID	DE	$I_{All}$	T-H-G	1-2

Note: T: Thermal, H: Hydro, G: Gas, PV: Photovoltaic, EV: Electrical vehicle, RES: Renewable energy sources, $I_{All}$: ITAE/ITSE/ISE/IAE.

**Table 3:** Mean and standard deviation for CEC 2017.

F.	D = 30		D = 50		D = 100	
	AHA	FDB-AHA	AHA	FDB-AHA	AHA	FDB-AHA
F1	5.93 × 10^3^ (6.25 × 10^3^)	**1.01 × 10^1^** **(7.20 × 10^1^)**	**4.02 × 10^3^** **(6.41 × 10^3^)**	5.34 × 10^3^ (7.97 × 10^3^)	1.08 × 10^4^ (1.18 × 10^4^)	**7.72 × 10^3^** **(1.09 × 10^4^)**
F3	1.71 (4.41)	**7.00 × 10^−8^** **(1.82 × 10^−7^)**	3.83 × 10^2^ (3.15 × 10^2^)	**8.55 × 10^−3^** **(1.33 × 10^−2^)**	1.03 × 10^4^ (3.60 × 10^3^)	**1.33 × 10^2^** **(9.23 × 10^1^)**
F4	8.38 × 10^1^ (3.13 × 10^1^)	**4.45 × 10^1^** **(3.81 × 10^1^)**	1.08 × 10^2^ (5.60 × 10^1^)	**1.03 × 10^2^** **(5.69 × 10^1^)**	2.63 × 10^2^ (5.55 × 10^1^)	**2.47 × 10^2^** **(4.08 × 10^1^)**
F5	1.27 × 10^2^ (3.07 × 10^1^)	**7.83 × 10^1^** **(1.95 × 10^1^)**	3.18 × 10^2^ (3.96 × 10^1^)	**1.95 × 10^2^** **(3.37 × 10^1^)**	7.98 × 10^2^ (6.75 × 10^1^)	**5.86 × 10^2^** **(7.78 × 10^1^)**
F6	2.37 (4.00)	**9.46 × 10^−2^** **(1.77 × 10^−1^)**	6.00 (8.79)	**6.23 × 10^−1^** **(6.80 × 10^−1^)**	1.40 × 10^1^ (9.94)	**1.66** **(1.36)**
F7	2.25 × 10^2^ (7.03 × 10^1^)	**1.38 × 10^2^** **(2.47 × 10^1^)**	6.06 × 10^2^ (1.74 × 10^2^)	**3.37 × 10^2^** **(8.65 × 10^1^)**	2.05 × 10^3^ (3.37 × 10^2^)	**1.37 × 10^3^** **(1.66 × 10^2^)**
F8	1.22 × 10^2^ (2.78 × 10^1^)	**7.31 × 10^1^** **(1.90 × 10^1^)**	3.37 × 10^2^ (4.42 × 10^1^)	**1.98 × 10^2^** **(3.49 × 10^1^)**	8.89 × 10^2^ (9.33 × 10^1^)	**5.93 × 10^2^** **(6.57 × 10^1^)**
F9	2.39 × 10^3^ (9.50 × 10^2^)	**4.41 × 10^2^** **(3.24 × 10^2^)**	8.62 × 10^3^ (2.68 × 10^3^)	**4.76 × 10^3^** **(1.64 × 10^3^)**	2.18 × 10^4^ (8.31 × 10^2^)	**1.68 × 10^4^** **(2.93 × 10^3^)**
F10	3.29 × 10^3^ (5.58 × 10^2^)	**2.58 × 10^3^** **(5.16 × 10^2^)**	5.43 × 10^3^ (9.90 × 10^2^)	**4.83 × 10^3^** **(6.85 × 10^2^)**	1.35 × 10^4^ (1.06 × 10^3^)	**1.19 × 10^4^** **(1.28 × 10^3^)**
F11	7.69 × 10^1^ (2.87 × 10^1^)	**5.75 × 10^1^** **(2.83 × 10^1^)**	1.32 × 10^2^ (2.63 × 10^1^)	**1.12 × 10^2^** **(3.11 × 10^1^)**	5.86 × 10^2^ (1.09 × 10^2^)	**4.05 × 10^2^** **(8.18 × 10^1^)**
F12	2.15 × 10^5^ (1.47 × 10^5^)	**5.94 × 10^4^** **(3.99 × 10^4^)**	1.59 × 10^6^ (7.63 × 10^5^)	**8.26 × 10^5^** **(4.68 × 10^5^)**	6.01 × 10^6^ (2.53 × 10^6^)	**2.28 × 10^6^** **(8.97 × 10^5^)**
F13	1.58 × 10^4^ (1.70 × 10^4^)	**1.08 × 10^4^** **(1.38 × 10^4^)**	**4.89 × 10^3^** **(6.16 × 10^3^)**	4.97 × 10^3^ (6.62 × 10^3^)	6.54 × 10^3^ (6.34 × 10^3^)	**5.33 × 10^3^** **(6.30 × 10^3^)**
F14	5.05 × 10^3^ (4.76 × 10^3^)	**1.65 × 10^3^** **(2.13 × 10^3^)**	4.25 × 10^4^ (2.65 × 10^4^)	**1.60 × 10^4^** **(1.31 × 10^4^)**	4.39 × 10^5^ (1.38 × 10^5^)	**1.34 × 10^5^** **(5.14 × 10^4^)**
F15	**2.60 × 10^3^** **(2.83 × 10^3^)**	5.34 × 10^3^ (8.11 × 10^3^)	9.96 × 10^3^ (6.59 × 10^3^)	**4.58 × 10^3^** **(4.91 × 10^3^)**	**1.71 × 10^3^** **(1.94 × 10^3^)**	3.44 × 10^3^ (4.67 × 10^3^)
F16	1.02 × 10^3^ (2.90 × 10^2^)	**9.54 × 10^2^** **(3.03 × 10^2^)**	**1.61 × 10^3^** **(3.93 × 10^2^)**	1.70 × 10^3^ (4.56 × 10^2^)	4.20 × 10^3^ (7.06 × 10^2^)	**4.06 × 10^3^** **(7.06 × 10^2^)**
F17	4.65 × 10^2^ (2.22 × 10^2^)	**4.02 × 10^2^** **(1.77 × 10^2^)**	1.43 × 10^3^ (3.12 × 10^2^)	**1.21 × 10^3^** **(2.62 × 10^2^)**	3.25 × 10^3^ (5.61 × 10^2^)	**3.00 × 10^3^** **(4.93 × 10^2^)**
F18	5.76 × 10^4^ (4.18 × 10^4^)	**1.86 × 10^4^** **(1.48 × 10^4^)**	2.19 × 10^5^ (1.11 × 10^5^)	**7.11 × 10^4^** **(3.04 × 10^4^)**	5.19 × 10^5^ (1.95 × 10^5^)	**2.33 × 10^5^** **(7.33 × 10^4^)**
F19	**3.72 × 10^3^** **(4.24 × 10^3^)**	6.41 × 10^3^ (9.03 × 10^3^)	1.45 × 10^4^ (9.33 × 10^3^)	**1.17 × 10^4^** **(8.65 × 10^3^)**	2.96 × 10^3^ (2.93 × 10^3^)	**2.43 × 10^3^** **(3.00 × 10^3^)**
F20	4.53 × 10^2^ (1.76 × 10^2^)	**4.30 × 10^2^** **(1.67 × 10^2^)**	1.11 × 10^3^ (3.60 × 10^2^)	**9.71 × 10^2^** **(3.35 × 10^2^)**	3.09 × 10^3^ (6.77 × 10^2^)	**2.80 × 10^3^** **(4.80 × 10^2^)**
F21	2.94 × 10^2^ (2.60 × 10^1^)	**2.77 × 10^2^** **(2.00 × 10^1^)**	4.28 × 10^2^ (3.43 × 10^1^)	**3.79 × 10^2^** **(3.58 × 10^1^)**	9.12 × 10^2^ (8.41 × 10^1^)	**7.97 × 10^2^** **(8.02 × 10^1^)**
F22	**1.01 × 10^2^** **(1.71)**	1.77 × 10^2^ (5.47 × 10^2^)	6.20 × 10^3^ (1.86 × 10^3^)	**5.50 × 10^3^** **(1.31 × 10^3^)**	1.69 × 10^4^ (1.62 × 10^3^)	**1.40 × 10^4^** **(1.10 × 10^3^)**
F23	4.75 × 10^2^ (3.16 × 10^1^)	**4.46 × 10^2^** **(2.64 × 10^1^)**	7.36 × 10^2^ (6.59 × 10^1^)	**6.56 × 10^2^** **(3.93 × 10^1^)**	1.02 × 10^3^ (8.09 × 10^1^)	**9.66 × 10^2^** **(7.53 × 10^1^)**
F24	5.76 × 10^2^ (4.11 × 10^1^)	**5.36 × 10^2^** **(2.89 × 10^1^)**	8.81 × 10^2^ (8.36 × 10^1^)	**8.13 × 10^2^** **(6.99 × 10^1^)**	1.86 × 10^3^ (1.70 × 10^2^)	**1.73 × 10^3^** **(1.35 × 10^2^)**
F25	4.00 × 10^2^ (1.86 × 10^1^)	**3.88 × 10^2^** **(5.19)**	5.75 × 10^2^ (3.26 × 10^1^)	**5.55 × 10^2^** **(3.82 × 10^1^)**	8.02 × 10^2^ (6.66 × 10^1^)	**7.95 × 10^2^** **(7.29 × 10^1^)**
F26	**1.22 × 10^3^** **(1.50 × 10^3^)**	1.78 × 10^3^ (8.53 × 10^2^)	**2.89 × 10^3^** **(3.30 × 10^3^)**	3.87 × 10^3^ (1.41 × 10^3^)	1.68 × 10^4^ (7.90 × 10^3^)	**1.36 × 10^4^** **(1.92 × 10^3^)**
F27	5.57 × 10^2^ (1.83 × 10^1^)	**5.33 × 10^2^** **(1.43 × 10^1^)**	9.00 × 10^2^ (1.38 × 10^2^)	**7.42 × 10^2^** **(9.43 × 10^1^)**	1.19 × 10^3^ (1.35 × 10^2^)	**9.83 × 10^2^** **(1.10 × 10^2^)**
F28	3.95 × 10^2^ (3.18 × 10^1^)	**3.67 × 10^2^** **(6.61 × 10^1^)**	5.06 × 10^2^ (3.29 × 10^1^)	**5.00 × 10^2^** **(2.85 × 10^1^)**	6.11 × 10^2^ (3.71 × 10^1^)	**5.90 × 10^2^** **(3.13 × 10^1^)**
F29	8.97 × 10^2^ (2.16 × 10^2^)	**7.46 × 10^2^** **(1.85 × 10^2^)**	1.45 × 10^3^ (3.58 × 10^2^)	**1.19 × 10^3^** **(2.82 × 10^2^)**	4.11 × 10^3^ (4.80 × 10^2^)	**3.75 × 10^3^** **(6.01 × 10^2^)**

Note: The first line represents the mean value, while the value in parentheses represents the standard deviation. Bold values indicate the best results.

**Table 4:** Mean and standard deviation for CEC 2020.

F.	D = 30		D = 50		D = 100	
	AHA	FDB-AHA	AHA	FDB-AHA	AHA	FDB-AHA
F1	4.15 × 10^3^ (5.93 × 10^3^)	**2.97 × 10^−4^** **(2.08 × 10^−3^)**	5.40 × 10^3^ (6.45 × 10^3^)	**5.16 × 10^3^** **(6.32 × 10^3^)**	**7.57 × 10^3^** **(7.05 × 10^3^)**	1.14 × 10^4^ (1.44 × 10^4^)
F2	2.95 × 10^3^ (5.16 × 10^2^)	**2.46 × 10^3^** **(5.70 × 10^2^)**	6.67 × 10^3^ (1.13 × 10^3^)	**4.91 × 10^3^** **(7.19 × 10^2^)**	1.37 × 10^4^ (1.22 × 10^3^)	**1.24 × 10^4^** **(1.23 × 10^3^)**
F3	2.14 × 10^2^ (7.19 × 10^1^)	**1.42 × 10^2^** **(3.28 × 10^1^)**	6.20 × 10^2^ (1.92 × 10^2^)	**3.53 × 10^2^** **(7.56 × 10^1^)**	2.01 × 10^3^ (3.32 × 10^2^)	**1.36 × 10^3^** **(1.85 × 10^2^)**
F4	0.00 (0.00)	0.00 (0.00)	0.00 (0.00)	0.00 (0.00)	0.00 (0.00)	0.00 (0.00)
F5	1.58 × 10^5^ (8.94 × 10^4^)	**3.54 × 10^4^** **(2.38 × 10^4^)**	5.40 × 10^5^ (2.66 × 10^5^)	**1.60 × 10^5^** **(8.16 × 10^4^)**	2.53 × 10^6^ (7.54 × 10^5^)	**1.34 × 10^6^** **(4.19 × 10^5^)**
F6	2.67 × 10^2^ (1.49 × 10^2^)	**1.90 × 10^2^** **(1.23 × 10^2^)**	7.52 × 10^2^ (2.43 × 10^2^)	**5.85 × 10^2^** **(1.90 × 10^2^)**	2.91 × 10^3^ (5.98 × 10^2^)	**2.42 × 10^3^** **(5.04 × 10^2^)**
F7	1.53 × 10^5^ (1.35 × 10^5^)	**3.55 × 10^4^** **(2.69 × 10^4^)**	3.74 × 10^5^ (1.62 × 10^5^)	**1.99 × 10^5^** **(1.10 × 10^5^)**	2.16 × 10^6^ (6.11 × 10^5^)	**9.92 × 10^5^** **(4.10 × 10^5^)**
F8	**1.01 × 10^2^** **(1.75)**	2.16 × 10^2^ (5.76 × 10^2^)	6.64 × 10^3^ (1.64 × 10^3^)	**5.64 × 10^3^** **(1.11 × 10^3^)**	1.69 × 10^4^ (1.86 × 10^3^)	**1.38 × 10^4^** **(1.57 × 10^3^)**
F9	5.82 × 10^2^ (5.32 × 10^1^)	**5.35 × 10^2^** **(3.51 × 10^1^)**	9.14 × 10^2^ (8.81 × 10^1^)	**8.15 × 10^2^** **(6.89 × 10^1^)**	1.83 × 10^3^ (1.60 × 10^2^)	**1.68 × 10^3^** **(1.13 × 10^2^)**
F10	4.02 × 10^2^ (1.92 × 10^1^)	**3.93 × 10^2^** **(1.48 × 10^1^)**	5.79 × 10^2^ (2.31 × 10^1^)	**5.42 × 10^2^** **(3.70 × 10^1^)**	7.99 × 10^2^ (5.63 × 10^1^)	**7.82 × 10^2^** **(6.62 × 10^1^)**

Note: The first line represents the mean value, while the value in parentheses represents the standard deviation. Bold values indicate the best results.

**Table 5:** Friedman rankings of the compared algorithms.

Algorithm	CEC 2017			CEC 2020			Mean Rank
	D = 30	D = 50	D = 100	D = 30	D = 50	D = 100	
FDB-AHA	**5.30**	**5.20**	**4.68**	**4.84**	5.17	**5.09**	**5.05**
HS	5.35	6.07	7.68	7.37	8.07	8.42	7.16
JS	5.51	5.57	6.02	5.65	5.60	6.14	5.75
CGO	5.69	5.74	5.72	5.05	5.18	5.59	5.50
TLBO	5.89	6.14	6.74	6.41	5.94	6.48	6.27
PO	6.06	5.47	5.69	5.49	**5.15**	5.35	5.54
ARO	6.33	6.12	5.78	6.02	5.80	5.87	5.99
MA	7.18	7.25	6.64	6.74	6.46	5.94	6.70
MRFO	7.19	7.15	6.43	6.30	6.48	6.00	6.59
AHA	7.25	7.01	6.64	7.05	7.38	6.73	7.01
DE	7.61	8.11	7.82	8.69	9.02	7.32	8.10
GWO	8.62	8.16	8.15	8.39	7.75	9.08	8.36

Note: Bold values indicate the best (lowest) mean ranks.

**Table 6:** Wilcoxon pairwise comparison results.

vs. FDB-AHA	CEC 2017			CEC 2020		
+/=/−	D = 30	D = 50	D = 100	D = 30	D = 50	D = 100
ARO	**1/12/16**	**2/11/16**	**0/12/17**	**0/5/5**	**1/5/4**	**0/5/5**
CGO	**11/6/12**	**8/11/10**	**7/7/15**	**2/5/3**	3/4/3	**3/3/4**
DE	**6/5/18**	**5/3/21**	**7/5/17**	**1/2/7**	**1/2/7**	**2/3/5**
GWO	**4/5/20**	**5/4/20**	**3/5/21**	**0/3/7**	**1/3/6**	**1/1/8**
HS	13/7/9	**9/6/14**	**7/2/20**	**2/2/6**	**1/1/8**	**2/1/7**
JS	10/10/9	**10/6/13**	**6/9/14**	**3/3/4**	6/2/2	**2/5/3**
PO	13/4/12	**12/6/11**	12/7/10	3/4/3	4/2/4	3/4/3
TLBO	**6/13/10**	**6/9/14**	**3/9/17**	**1/4/5**	2/7/1	**2/4/4**
MA	**5/6/18**	**4/6/19**	**5/7/17**	**2/2/6**	**3/1/6**	**2/3/5**
MRFO	**2/10/17**	**3/9/17**	**4/11/14**	**1/4/5**	**1/5/4**	**2/3/5**

Note: Bold values indicate cases in which FDB-AHA is superior to the compared algorithm.

**Table 7:** Optimal controller parameters for System 1.

Controller Type	$K_{P1}$	$K_{P2}$	$K_{I}$	$K_{D}$	λ	μ
PID	1.0963	–	1.9999	0.4002	–	–
FOPID	2.2059	–	2.9999	0.5980	0.9999	1.0563
FOPI-FOPD	2.2992	2.9999	2.9999	0.4764	0.9991	1.1990

**Table 8:** ITAE and settling times for System 1.

Controller Type	ITAE	Settling Time (s)		
		$∆f_{1}$	$∆f_{2}$	** $∆P_{\mathrm{tie}}$ **
HSCOA: PID [34]	0.1247	2.36	2.75	2.42
PSO: PID [44]	0.4854	5.30	6.41	5.03
GA: PID [44]	0.4967	6.93	6.74	4.87
TLBO: PID [35]	0.2452	4.84	5.13	3.03
FDB-AHA: PID	0.1305	2.65	2.75	2.48
FDB-AHA: FOPID	0.0684	2.17	2.42	2.04
FDB-AHA: FOPI-FOPD	**0.0190**	**0.45**	**1.77**	**1.09**

Note: Bold values indicate the best results. The settling-time criterion is based on a ±0.002 band.

**Table 9:** FOPI-FOPD robustness analysis for System 1.

Parameter	Change Rate	Settling Time (s)			Undershoot (p.u.)		
		$∆f_{1}$	$∆f_{2}$	** $∆P_{\mathrm{tie}}$ **	$∆f_{1}$	$∆f_{2}$	** $∆P_{\mathrm{tie}}$ **
Nominal	-	0.45	1.77	1.09	−0.0476	−0.0154	−0.0052
$T_{g}$	+50%	0.98	1.53	1.14	−0.0556	−0.0195	−0.0062
	+25%	0.66	1.76	1.10	−0.0518	−0.0174	−0.0057
	−25%	0.56	1.79	1.10	−0.0430	−0.0136	−0.0048
	−50%	0.62	1.80	1.12	−0.0379	−0.0124	−0.0046
$T_{t}$	+50%	0.89	1.68	0.76	−0.0590	−0.0224	−0.0072
	+25%	0.81	1.68	0.98	−0.0536	−0.0189	−0.0062
	−25%	0.73	1.85	1.17	−0.0409	−0.0119	−0.0042
	−50%	0.91	1.94	1.25	−0.0330	−0.0091	−0.0037
$T_{12}$	+50%	0.78	1.59	1.02	−0.0465	−0.0199	−0.0067
	+25%	0.42	1.66	1.05	−0.0471	−0.0178	−0.0060
	−25%	0.50	1.93	1.16	−0.0482	−0.0126	−0.0043
	−50%	0.58	2.20	1.19	−0.0489	−0.0093	−0.0032
*B*	+50%	0.58	1.58	1.17	−0.0375	−0.0074	−0.0037
	+25%	0.51	1.68	1.14	−0.0418	−0.0104	−0.0043
	−25%	1.05	1.85	0.92	−0.0559	−0.0245	−0.0065
	−50%	2.06	2.46	1.36	−0.0686	−0.0435	−0.0083
*R*	+50%	0.47	1.71	1.06	−0.0478	−0.0158	−0.0053
	+25%	0.46	1.74	1.08	−0.0477	−0.0156	−0.0052
	−25%	0.44	1.82	1.12	−0.0474	−0.0150	−0.0051
	−50%	0.42	1.92	1.16	−0.0471	−0.0142	−0.0050

Note: The settling-time criterion is based on a ±0.002 band.

**Table 10:** Optimal controller parameters for System 2.

Controller Type	Source	$K_{P1}$	$K_{P2}$	$K_{I}$	$K_{D}$	λ	μ
PID	T	1.9999	–	0.4906	0.8370	–	–
	H	1.9999	–	−0.1509	0.0992	–	–
	G	1.7587	–	1.9999	1.9898	–	–
FOPID	T	1.9862	–	1.9992	1.9838	0.5966	0.0028
	H	−0.1838	–	1.7499	−1.7899	0.9272	0.2356
	G	1.9941	–	1.9991	1.9624	0.9965	1.1321
FOPI-FOPD	T	−1.9997	−1.9972	−1.9975	−1.9572	0.6413	0.6620
	H	1.2403	−1.9237	−1.8164	0.4744	0.9481	1.4776
	G	−1.3845	−1.9999	−1.9989	−1.9669	0.9496	0.0342

Note: T: Thermal, H: Hydro, G: Gas.

**Table 11:** ITAE and settling times for System 2.

Controller Type	ITAE	Settling Time (s)		
		$∆f_{1}$	$∆f_{2}$	** $∆P_{\mathrm{tie}}$ **
LUS-TLBO: PID [37]	0.2130	2.27	2.81	1.94
DE: PID [45]	0.5283	6.74	4.61	4.32
PID	0.1471	3.09	4.06	1.81
FOPID	0.0630	1.36	2.14	1.45
FOPI-FOPD	**0.0206**	**0.68**	**1.20**	**0**

Note: Bold values indicate the best results. The settling-time criterion is based on a ±0.002 band.

**Table 12:** FOPI-FOPD robustness analysis for System 2.

Parameter	Change Rate	Settling Time (s)			Undershoot (p.u.)		
		$∆f_{1}$	$∆f_{2}$	$∆P_{\mathrm{tie}}$	$∆f_{1}$	$∆f_{2}$	** $∆P_{\mathrm{tie}}$ **
Nominal	–	0.68	1.20	0	−0.0131	−0.0050	−0.0013
$T_{g}$	+50%	1.55	2.05	0	−0.0142	−0.0057	−0.0014
	+25%	1.45	1.87	0	−0.0137	−0.0054	−0.0014
	−25%	0.70	1.26	0	−0.0126	−0.0047	−0.0013
	−50%	0.75	1.43	0	−0.0119	−0.0043	−0.0012
$T_{t}$	+50%	1.18	1.26	0	−0.0148	−0.0064	−0.0016
	+25%	0.71	1.23	0	−0.0141	−0.0057	−0.0015
	−25%	1.26	1.72	0	−0.0121	−0.0042	−0.0012
	−50%	1.18	1.71	0	−0.0107	−0.0034	−0.0010
$T_{12}$	+50%	1.45	1.91	0	−0.0129	−0.0066	−0.0017
	+25%	1.42	1.86	0	−0.0130	−0.0059	−0.0015
	−25%	0.73	1.26	0	−0.0133	−0.0040	−0.0011
	−50%	0.80	1.25	0	−0.0135	−0.0029	−0.0008
*B*	+50%	1.12	0.77	0	−0.0115	−0.0027	−0.0009
	+25%	1.21	0.97	0	−0.0122	−0.0036	−0.0011
	−25%	1.66	1.41	0	−0.0147	−0.0076	−0.0016
	−50%	2.96	3.56	0.84	−0.0171	−0.0126	−0.0021
*R*	+50%	0.70	1.24	0	−0.0132	−0.0051	−0.0013
	+25%	0.69	1.23	0	−0.0132	−0.0051	−0.0013
	−25%	1.32	1.16	0	−0.0132	−0.0050	−0.0013
	−50%	1.42	1.86	0	−0.0132	−0.0048	−0.0013

Note: The settling-time criterion is based on a ±0.002 band.

**Table 13:** Optimal controller parameters for System 3.

Controller Type	Source	$K_{P1}$	$K_{P2}$	$K_{I}$	$K_{D}$	λ	μ
PID	PV	0.0406	–	0.0129	0.0654	–	–
	T	1.9999	–	1.9999	0.5629	–	–
FOPID	PV	0.3203	–	0.0214	1.1113	1.3042	1.4656
	T	1.9329	–	1.9414	0.5658	0.8823	0.8232
FOPI-FOPD	PV	0.6207	0.1439	0.1799	1.7302	1.3568	1.3601
	T	1.9999	1.9999	2	0.8903	1.3015	0.6920

Note: T: Thermal; PV: Photovoltaic.

**Table 14:** ITAE and settling times for System 3.

Controller Type	ITAE	Settling Time (s)		
		$∆f_{1}$	$∆f_{2}$	** $∆P_{\mathrm{tie}}$ **
MA: PID [51]	0.7577	8.76	5.97	11.66
MA: PI-PD [51]	0.3379	3.79	**1**	8.64
FDB-AHA: PID	0.7577	8.75	5.96	11.60
FDB-AHA: FOPID	0.5945	**2.82**	5.29	11.02
FDB-AHA: FOPI-FOPD	**0.2413**	6.22	5.76	**1.58**

Note: Bold values indicate the best results for each metric. The settling-time criterion is based on a ±0.002 band.

**Table 15:** FOPI-FOPD robustness analysis for System 3.

Parameter	Change Rate	Settling Time (s)			Undershoot (pu)		
		$∆f_{1}$	$∆f_{2}$	** $∆P_{\mathrm{tie}}$ **	$∆f_{1}$	$∆f_{2}$	** $∆P_{\mathrm{tie}}$ **
Nominal	–	6.22	5.76	1.58	−0.0790	−0.1012	−0.0019
$T_{g}$	+50%	6.20	5.72	1.64	−0.0882	−0.1123	−0.0019
	+25%	6.20	5.75	1.60	−0.0838	−0.1069	−0.0019
	−25%	6.25	5.79	1.57	−0.0738	−0.0949	−0.0019
	−50%	6.27	5.81	1.59	−0.0685	−0.0881	−0.0019
$T_{t}$	+50%	6.04	5.56	1.08	−0.0947	−0.1193	−0.0019
	+25%	6.13	5.67	1.58	−0.0872	−0.1107	−0.0019
	−25%	6.32	5.86	1.59	−0.0696	−0.0903	−0.0019
	−50%	6.40	5.95	1.69	−0.0587	−0.0773	−0.0018
$T_{12}$	+50%	6.10	5.79	1.55	−0.0853	−0.1003	−0.0019
	+25%	6.15	5.78	1.56	−0.0827	−0.1007	−0.0019
	−25%	6.34	5.75	1.60	−0.0731	−0.1019	−0.0018
	−50%	6.55	5.73	1.64	−0.0632	−0.1028	−0.0017
*B*	+50%	5.82	5.48	1.26	−0.0633	−0.0833	−0.0013
	+25%	6.02	5.62	1.41	−0.0700	−0.0909	−0.0015
	−25%	6.42	5.89	5.48	−0.0920	−0.1159	−0.0024
	−50%	6.54	5.97	6.39	−0.1130	−0.1392	−0.0032
*R*	+50%	6.13	5.68	1.57	−0.0796	−0.1018	−0.0019
	+25%	6.17	5.71	1.58	−0.0793	−0.1016	−0.0019
	−25%	6.32	5.85	1.57	−0.0784	−0.1006	−0.0018
	−50%	6.49	6.01	1.57	−0.0774	−0.0995	−0.0018

Note: The settling-time criterion is based on a ±0.002 band.

**Table 16:** MSE values between MATLAB and OPAL-RT responses.

Controller	$∆f_{1}$	$∆f_{2}$	$∆P_{\mathrm{tie}}$
PID	$2.6438\times 10^{-13}$	$6.8382\times 10^{-14}$	$6.8139\times 10^{-15}$
FOPID	$2.7068\times 10^{-11}$	$7.3370\times 10^{-12}$	$1.0858\times 10^{-12}$
FOPI-FOPD	$1.8256\times 10^{-9}$	$2.0312\times 10^{-10}$	$2.4295\times 10^{-11}$

## Data Availability

Data are available on request from the corresponding author.

## Appendix A. Power System Parameters

System 1 [49]:

$P_{R}=2000\;\mathrm{MW},\;P_{L}=1000\;\mathrm{MW},\;f=60\;\mathrm{Hz},\;R_{1}=R_{2}=2.4\;\mathrm{Hz}/\mathrm{pu},\;B_{1}=B_{2}=\;0.425\;\mathrm{pu}\;\mathrm{MW}/\mathrm{Hz},\;K_{PS}=120\;\mathrm{Hz}/\mathrm{pu},\;T_{ps}=20\;s,\;T_{g}=0.08\;s,\;T_{t}=0.3\;s,\;T_{12}=0.545\;\mathrm{pu}.$

System 2 [31]:

$R_{1}=R_{2}=2.4\;\mathrm{Hz}/\mathrm{pu},\;B_{1}=B_{2}=0.4312\;\mathrm{pu}\;\mathrm{MW}/\mathrm{Hz},\;K_{PS}=68.9566\;\mathrm{Hz}/\mathrm{pu},\;T_{ps}=\;11.49\;s,\;T_{12}=0.0433\;\mathrm{pu},\;T_{sg}=0.08\;s,\;T_{t}=0.3\;s,\;K_{r}=0.3,\;T_{r}=10\;s,\;K_{T}=\;0.543478,\;T_{gh}=0.2\;s,\;T_{RS}=5\;s,\;T_{rh}=28.75\;s,\;T_{w}=1\;s,\;K_{H}=0.326084,\;b_{g}=\;0.05\;s,\;c_{g}=1,\;X_{c}=0.6\;s,\;Y_{c}=1\;s,\;T_{cr}=0.01\;s,\;T_{f}=0.23\;s,\;T_{cd}=0.2\;s,\;K_{G}=0.130438.$

System 3 [51]:

$R=2.5\;\mathrm{Hz}/\mathrm{pu},\;B=0.8\;\mathrm{pu}\;\mathrm{MW}/\mathrm{Hz},\;K_{PS}=120\;\mathrm{Hz}/\mathrm{pu},\;T_{ps}=20\;s,\;T_{12}=\;0.545\;\mathrm{pu},\;T_{g}=0.08\;s,\;T_{t}=0.3\;s,\;K_{r}=0.33,\;T_{r}=10\;s,\;\alpha =900,\;b=-18,\;c=\;100,\;d=50.$
